# Microbubble cavitation restores *Staphylococcus aureus* antibiotic susceptibility in vitro and in a septic arthritis model

**DOI:** 10.1038/s42003-023-04752-y

**Published:** 2023-04-17

**Authors:** Neil Zhao, Dylan Curry, Rachel E. Evans, Selin Isguven, Theresa Freeman, John R. Eisenbrey, Flemming Forsberg, Jessica M. Gilbertie, Sophie Boorman, Rachel Hilliard, Sana S. Dastgheyb, Priscilla Machado, Maria Stanczak, Marc Harwood, Antonia F. Chen, Javad Parvizi, Irving M. Shapiro, Noreen J. Hickok, Thomas P. Schaer

**Affiliations:** 1 Department of Orthopaedic Surgery, Sidney Kimmel College, Thomas Jefferson University, Philadelphia, PA USA; 2 Department of Radiology, Thomas Jefferson University, Philadelphia, PA USA; 3 Department of Clinical Studies, New Bolton Center, School of Veterinary Medicine, University of Pennsylvania, Kennett Square, PA USA; 4 Rothman Orthopaedic Institute, Philadelphia, PA USA; 5 Department of Orthopaedic Surgery, Brigham and Women’s Hospital, Harvard Medical School, Boston, MA USA

**Keywords:** Infectious diseases, Biofilms

## Abstract

Treatment failure in joint infections is associated with fibrinous, antibiotic-resistant, floating and tissue-associated *Staphylococcus aureus* aggregates formed in synovial fluid (SynF). We explore whether antibiotic activity could be increased against *Staphylococcus aureus* aggregates using ultrasound-triggered microbubble destruction (UTMD), in vitro and in a porcine model of septic arthritis. In vitro, when bacterially laden SynF is diluted, akin to the dilution achieved clinically with lavage and local injection of antibiotics, amikacin and ultrasound application result in increased bacterial metabolism, aggregate permeabilization, and a 4-5 log decrease in colony forming units, independent of microbubble destruction. Without SynF dilution, amikacin + UTMD does not increase antibiotic activity. Importantly, in the porcine model of septic arthritis, no bacteria are recovered from the SynF after treatment with amikacin and UTMD—ultrasound without UTMD is insufficient. Our data suggest that UTMD + antibiotics may serve as an important adjunct for the treatment of septic arthritis.

## Abstract (web summary)

Coupling ultrasound-triggered microbubble destruction with antibiotics in diluted synovial fluid leads to the effective clearance of S. aureus in models of septic arthritis

## Introduction

Biofilms, whether floating or adhesive, are assumed to be the major cause of infections, and treatment is made difficult by the increased antibiotic tolerance associated with the bacteria that constitute these biofilms^1^. This problem is exemplified in joint infections, where despite aggressive local and systemic antibiotic treatments, infections can recur in up to 45% of septic arthritis cases^2^. *Staphylococcus aureus* (*S. aureus*) is the causative organism in as many as 42% of these cases of septic arthritis^3^, a debilitating disease that has an 11% mortality rate^4^, affects ~20,000 people annually in the United States^5^ with medical expenses >$1 billion yearly^6^. *S. aureus* aggregates in synovial fluid (SynF)^7–12^, the lubricious fluid within the joint, and can be sequestered within different joint compartments as well as the fibrin-rich pannus, so that joint drainage is often insufficient for their removal^13^. Current treatment options include joint lavage/drainage, aggressive local and systemic antibiotic intervention, and surgical debridement. The continued presence of these aggregates is thought to be a cause of treatment failure^14^. Thus, we explored strategies for restoring antibiotic susceptibility of bacterial aggregates to improve the outcome of acute septic arthritis treatments.

Biofilms are three-dimensional bacterial communities rich in proteins, polysaccharides, and extracellular DNA (eDNA)^15^. Reduced antibiotic penetration, slowed metabolism resulting from adaptive changes to a nutrient-depleted environment, and the emergence of persister cells make biofilms tolerant to antibiotics. Unlike many biofilms which can be dispersed with DNase or dispersin B, SynF bacterial aggregates do not disperse with these treatments and require aggressive enzymatic digestion, e.g., proteinase K or tissue plasminogen activator (TPA)^7,10,16–18^. The use of proteinase K in a physiological site could cause tissue damage^19^ and the effects of activation of plasminogen by TPA would need to be delineated. We, therefore, turned to mechanical means, specifically, ultrasound-triggered microbubble destruction (UTMD) to perturb aggregate structure. Microbubbles, typically 1–8 µm in diameter, are ultrasound contrast agents that are comprised of a polymer, protein, or lipid shell surrounding a gas core^20^. UTMD uses ultrasound waves to cause inertial cavitation of microbubbles, producing shockwaves emanating from the microbubbles. UTMD causes simple in vitro biofilms and catheter-associated biofilms to become more susceptible to vancomycin^21,22^. Other phase contrast agents of smaller diameters have also been used to enhance antibiotic efficacy against biofilms^23^. However, ultrasound effects on septic joints and SynF bacterial aggregates are limited to a single equine case study without the use of the augmentation associated with UTMD^24^. We hypothesize that UTMD will: (1) permeabilize *S. aureus* aggregates to allow for antibiotic penetration, and (2) restore bacterial metabolic activity. Both would increase antibiotic susceptibility.

We investigated the conditions under which UTMD reproducibly increased the amikacin (AMK) susceptibility of bacterial aggregates in SynF, in vitro. AMK was chosen as it is a standard antibiotic used in the treatment of veterinary septic joints^25–28^ with human usage predominantly in neonates^29,30^. We identified bacterial response to the synovial fluid environment and conditions which resulted in markedly increased *S. aureus* aggregate susceptibility to antibiotics, in vitro. We used similar conditions in a study of septic arthritis in pigs. Based on the results of this study, we propose UTMD combined with antibiotics as an effective, inexpensive, and readily accessible method for the reduction of bacterial load, especially with regard to the antibiotic-tolerant aggregates that are thought to be a major barrier to successful treatment of septic arthritis. We suggest that UTMD + antibiotics allows increased antibiotic activity in infections associated with floating biofilms and could be used as an adjunct to the systemic antibiotic treatments that are standard for the treatment of septic joints.

## Results

### Aggregation suppresses metabolic activity

**Fig. 1:**
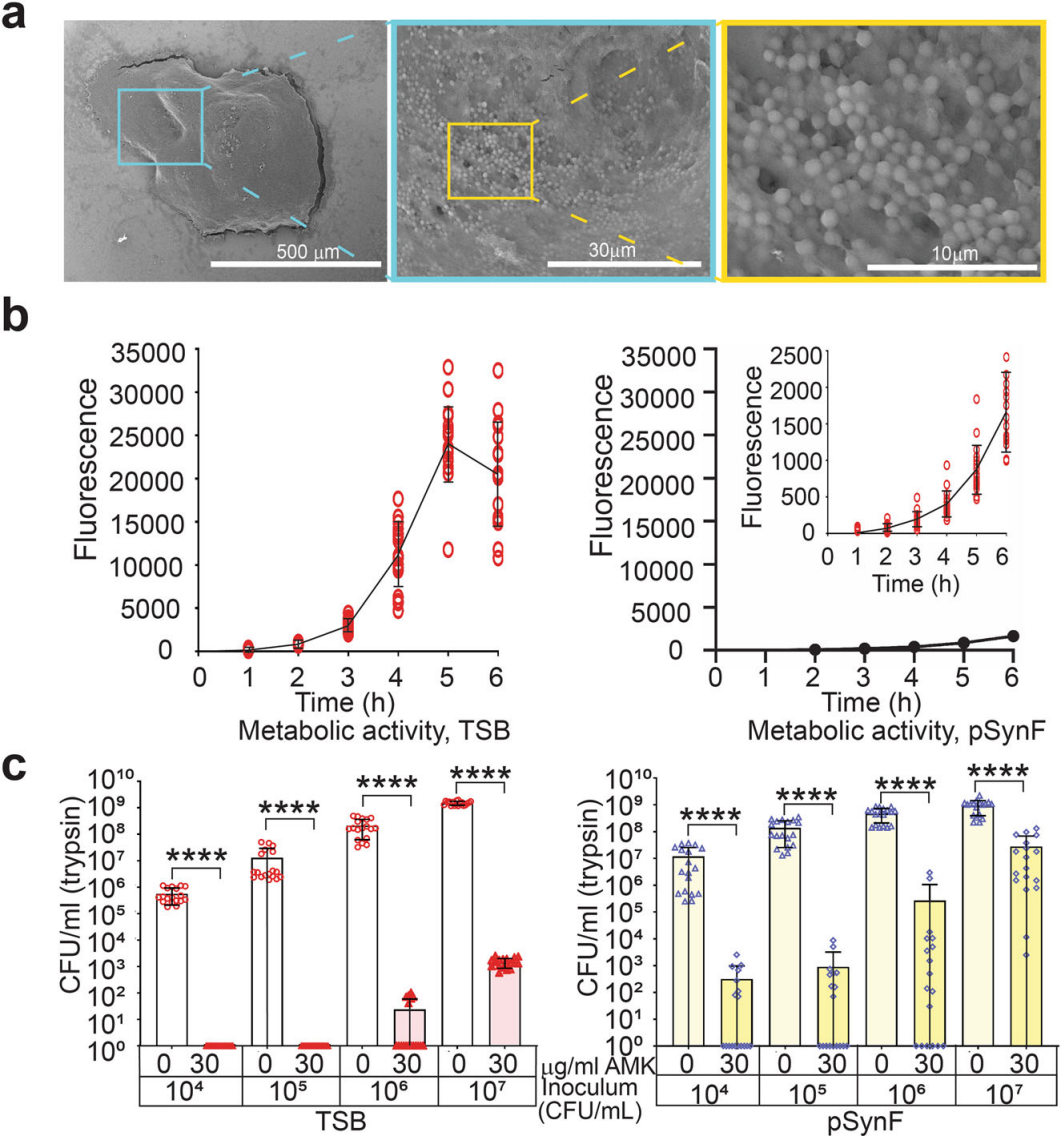
*S. aureus* aggregates in pSynF are metabolically suppressed and exhibit tolerance to amikacin. **a** Scanning electron micrographs of an *S. aureus* aggregate formed in a model SynF, i.e., pSynF. Successive magnifications are indicated and highlight the bacteria covered in matrix. **b** Alamar blue fluorescence as an indicator of *S. aureus* metabolic activity in TSB and pSynF (inset shows an expansion of the y-axis). Note the >10× suppression in metabolic activity in pSynF. **c** *S. aureus* sensitivity to 30 µg/mL of AMK as a function of initial inoculum and of culturing in TSB or pSynF. Notably, bacterial survival is apparent even at the lowest inoculum when cultured in pSynF. Data presented as mean ± standard deviation. For each point **b** or bar **c**, *n* = 18. Individual data points are shown as open, colored symbols. Normality checked using Kolmogorov–Smirnov test. Comparisons done using two-tailed Wilcoxon rank sum test. ****P < 0.0001.

Because antibiotic action is predicated on the inhibition of functions of dividing cells, we first investigated whether antibiotic tolerance of bacteria in SynF was due to the induction of metabolic quiescence. In human SynF and in a recently described pseudo-synovial fluid (pSynF)^8^, methicillin-susceptible *S. aureus* (MSSA) aggregate into free-floating, dense, fibrous, structures that have characteristics of antibiotic-tolerant biofilms (Fig. 1a, increasing magnifications show the clustered bacteria within the aggregate; these bacteria are encased in a matrix). MSSA in pSynF showed ~1/10 the metabolic rate of that in trypticase soy broth (TSB) at 6 h, as measured by alamar blue fluorescence (pSynF) (Fig. 1b). This repressed metabolism resulted in decreased antibiotic susceptibility. Specifically, 30 μg/mL amikacin (AMK) reduced MSSA counts (Fig. 1c) by 6–7 logs (*p* < 0.0001) in TSB at 6 h. Overall, antibiotic tolerance was greater in pSynF (Fig. 1c), in keeping with reduced metabolism. Antibiotic tolerance increased in both TSB and pSynF as the inoculum increased. In TSB, some bacterial survival occurred in the higher inoculum, but the overall decrease remained constant. However, in pSynF, antibiotic tolerance increased with increasing inoculum. At 6 h, 30 μg/mL AMK reduced bacterial number by 4–5 logs at a starting inoculum of 10^4^ or 10^5^ CFU/mL, 3–4 logs at 10^6^ CFU/mL, and only 1–2 logs at 10^7^ CFU/mL (*p* < 0.0001). Thus, in pSynF, even a 10^4^ CFU/mL inoculum showed some survival after antibiotic challenge.

### Bath sonication causes small increases in aggregate dispersal and only minimal increases in antibiotic susceptibility

**Fig. 2:**
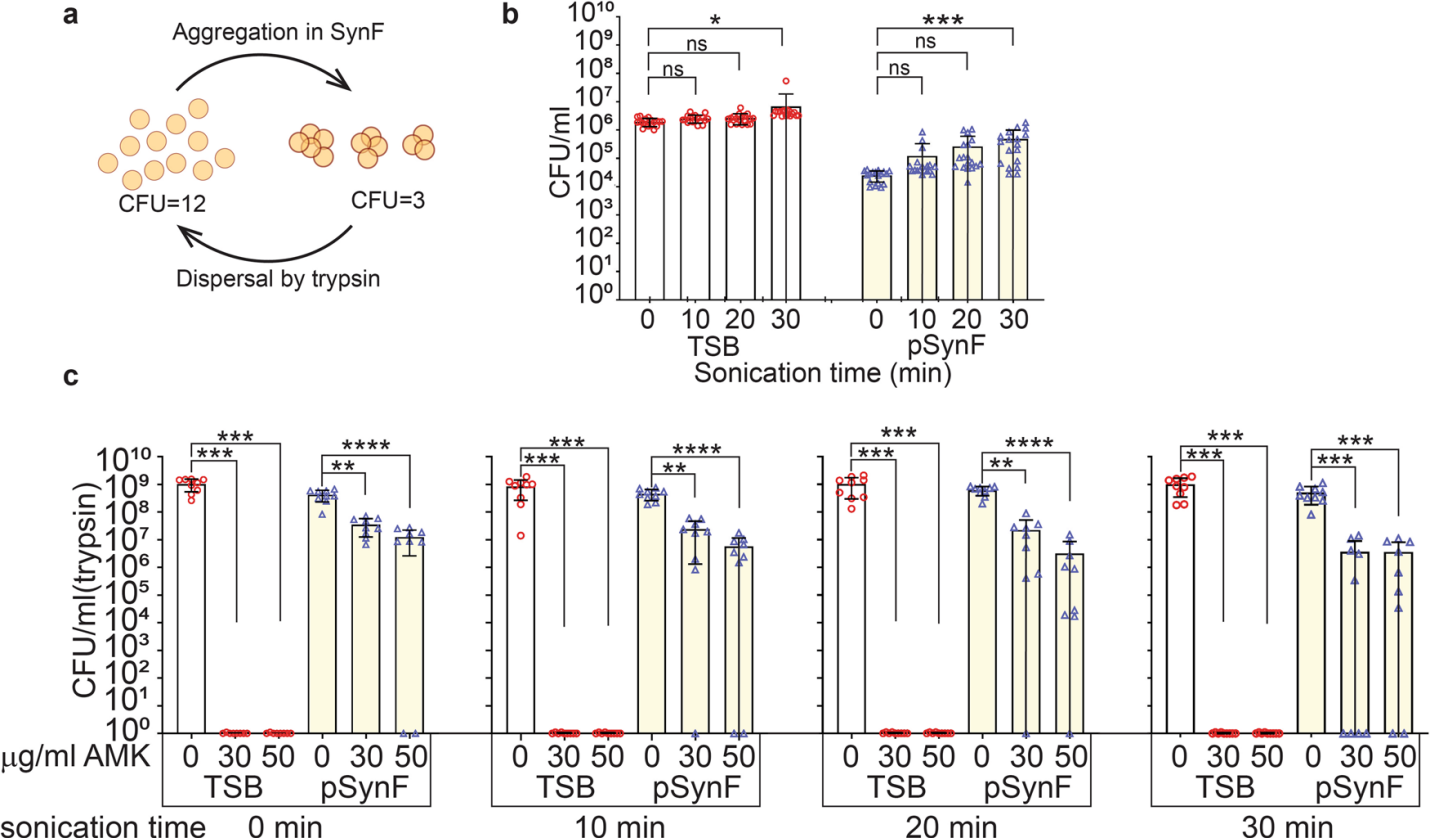
Water bath sonication causes slight dispersal of aggregates, with minimal increases in antibiotic sensitivity. **a** Diagram of *S. aureus* aggregation and dispersal demonstrating that aggregation causes a decrease in CFU. With dispersal, CFU (trypsin) approximates the CFU that would be obtained in a non-aggregating medium. **b** MSSA CFUs recovered in TSB or pSynF after increasing times of sonication in a water bath. TSB is presented as a control. The 30 min sonication results in the greatest dispersal of aggregates (increased CFU/ml). **c** Antibiotic (AMK) sensitivity of MSSA cultures in TSB or pSynF that were sonicated in a water bath sonicator for up to 30 min. Data are presented as mean ± standard deviation. Symbols indicate individual points and scatter of data. For each bar **b**, *n* = 17 (TSB, 20 min, 30 min; pSynF, 10 min, 30 min) or 18 (TSB, 0 min, 10 min; pSynF, 0 min, 20 min), or **c**, *n* = 9. Individual data points are shown as open, colored symbols. Normality was checked using Kolmogorov–Smirnov test. Comparisons were done using **b** ordinary ANOVA for multiple comparisons or **c** Kruskal–Wallis test for multiple comparisons. ns, *P* > 0.05; *P < 0.05; **P < 0.01; ***P < 0.001; ****P < 0.0001.

MSSA aggregation is detected by a decrease in recovered CFUs; when treated with trypsin (CFU/ml(trypsin), bacteria are dispersed allowing comparisons between media (Fig. 2a). Water bath sonication of MSSA in TSB at 90 min after inoculation (*t* = 0) caused a small increase (~0.5 log) in CFU/mL at 30 min (*p* = 0.0450) (Fig. 2b). In pSynF in which bacterial aggregation would occur during this 90 min pre-incubation, the effect of bath sonication was more pronounced with a ~1.5 log increase in CFU/mL after 30 min (*p* = 0.0002; Fig. 2b).

We investigated whether sonication increased antibiotic sensitivity (Fig. 2c). Independent of sonication, 30 or 50 μg/mL AMK in TSB eradicated MSSA (9 logs; *p* = 0.0002). In pSynF without sonication, AMK showed a dose-dependent effect (Fig. 2c), where 30 μg/mL AMK caused an ~1 log decrease (*p* = 0.0079) and 50 μg/mL AMK caused an ~1.5 log decrease (*p* < 0.0001) in CFU/mL(trypsin). The log decrease for the 30 μg/mL AMK treatment became slightly larger with sonication i.e., ~1.5 log for 10 (*p* = 0.0044) and 20 min (*p* = 0.0047), and ~2 log (*p* = 0.0004) for 30 min. After sonication of various lengths (10, 20, and 30 min), the log decrease in pSynF incubated with 50 μg/mL AMK remained at 1.5-2 logs (*p* < 0.0001, *p* < 0.0001, and *p* = 0.0009, respectively). The large error bars suggest that the overall response of MSSA to sonication was heterogeneous and that antibiotic susceptibility of MSSA was only minimally increased with sonication.

### Sonication with dilution increases metabolism and antibiotic susceptibility

**Fig. 3:**
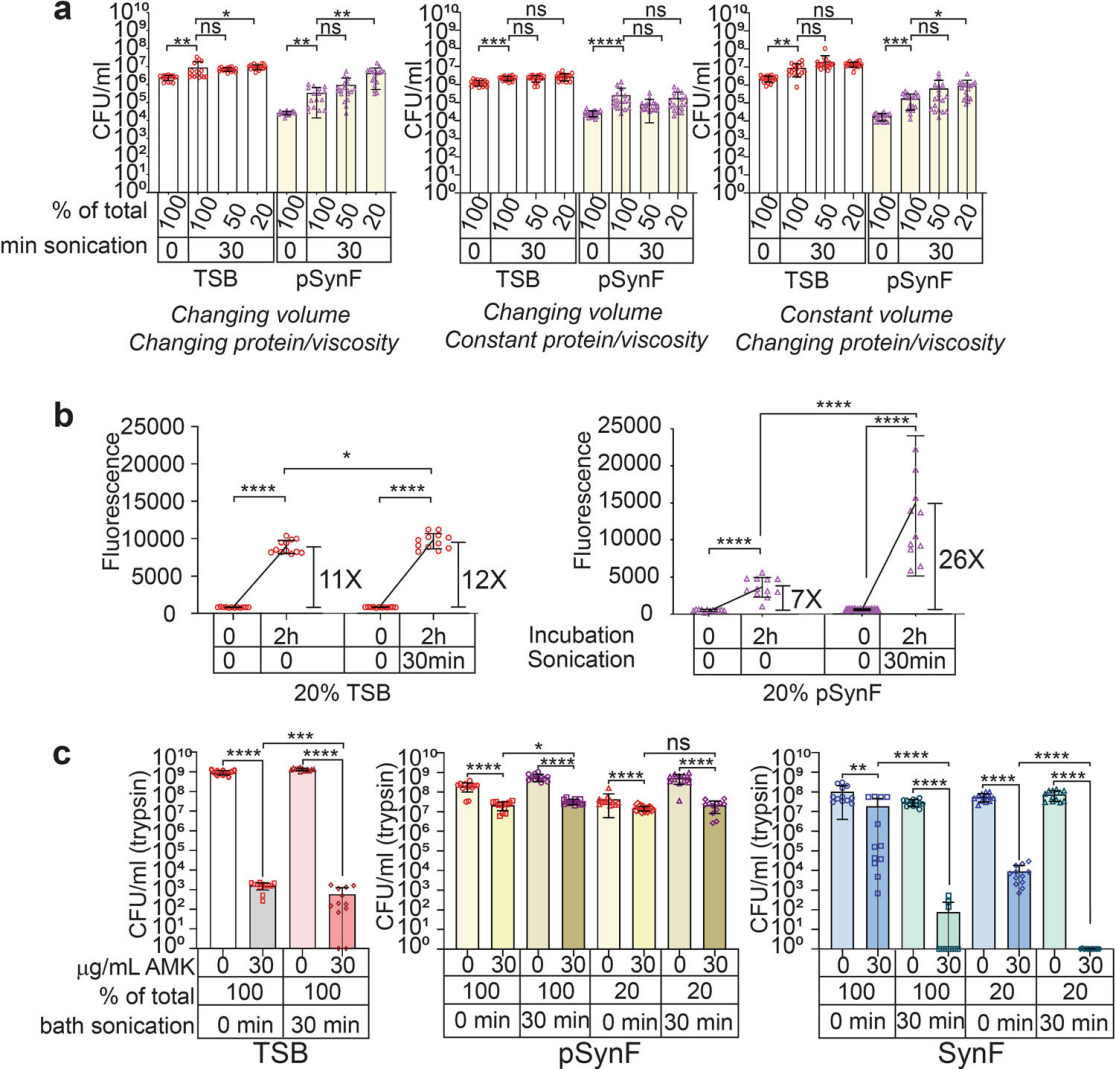
Dilution and water bath sonication increase MSSA AMK sensitivity. **a** The effects of increasing dilution on aggregate dispersal, where dilution with PBS (changing volume, changing protein/viscosity), pSynF (changing volume, constant viscosity), and PBS (constant volume, changing protein/viscosity) are indicated. **b** Changes in metabolic activity of *S. aureus* in 20% TSB and 20% pSynF with aggregated *S. aureus*, as indicated by Alamar blue fluorescence after bath sonication. **c** The effect of dilution and 30 min sonication on AMK sensitivity of MSSA in TSB, pSynF, and SynF. Data are presented as mean ± standard deviation. For each bar **a**, *n* = 16 (changing volume, changing viscosity; pSynF, 100%, 30 min) or 18, **b**, **c**, *n* = 12. Individual data points are shown as open, colored symbols. Normality was checked using Kolmogorov–Smirnov test. Comparisons were done using **a** Kruskal–Wallis test for multiple comparisons or (**b, c**) Mann–Whitney tests for paired comparison on the basis of independent groups. ns, *P* > 0.05; *P < 0.05; **P < 0.01; ***P < 0.001; ****P < 0.0001.

Joint lavage through arthroscopy is the minimum standard for treating septic arthritis and consists of a washout of SynF (often with saline), potential placement of local antibiotics, and ultimately a serosanguinous fluid replacing the saline lavage fluid^13,31^. To model treatment immediately following arthroscopy, where a combined saline/SynF mix would be present in the joint, we diluted TSB or pSynF after inoculation/aggregate formation and determined the effects of sonication. Without dilution (changing volume, changing protein/viscosity, Supplementary Table S1), sonication slightly increased CFU/mL in TSB (*p* = 0.0046) (Fig. 3a). Dilution to 50% or 20% TSB followed by sonication either had no additional effect or caused another slight increase in retrieved counts (*p* = 0.01625 compared to sonicated TSB). When pSynF and its dilutions (containing pre-formed MSSA aggregates) were sonicated, 100% pSynF showed an ~1 log increase in CFU/mL (*p* = 0.0031). 50% SynF did not further change with sonication, but 20% pSynF showed an additional increase in recovered counts (*p* < 0.0017). To test the individual effects of changes in total volume or protein concentration/viscosity, we first used TSB or pSynF as the dilutant to keep the protein/viscosity constant, but changed the volume (Fig. 3a; Supplementary Table S2). Recovered S. aureus in TSB and pSynF showed the expected increase in counts after sonication *p* = 0.001, *p* < 0.0001, respectively); further dilution/volume increases using the same medium did not alter dispersal. Finally, we kept the volume constant, but allowed the protein/viscosity to change by dilution with PBS to a fixed volume (Fig. 3a; Supplementary Tables S3, S4). CFU/mL in both TSB and pSynF showed small increases after sonication, as per previous graphs (*p* = 0.0015, *p* = 0.0004, respectively). No significant changes were observed in dispersal with the diluted TSB samples and only a small increase at 20% dilution in the pSynF sample (~1–1.5 log, *p* = 0.0397), suggesting that increased dispersal from aggregates depends on multiple factors including the protein/viscosity characteristics.

Because sonication of the diluted samples increased recovered counts in the pSynF samples, we investigated the effects of sonication and dilution on bacterial metabolism. Using 20% TSB, metabolism increased over a 2 h incubation (Fig. 3b) which was only slightly altered by the application of ultrasound (11-fold vs. 12-fold; *p* = 0.0449). In 20% pSynF, the effect of 2 h incubation on metabolism was less marked (sevenfold increase, *p* < 0.0001). After sonication, however, the metabolism of the diluted (20%) pSynF increased up to 26-fold (*p* < 0.0001), which was a significant increase over the unsonicated values (*p* < 0.0001), and metabolic levels approximated values measured in TSB.

We asked if these changes in metabolism and dispersion resulted in increased antibiotic sensitivity (Fig. 3c). In TSB, 30 μg/mL AMK caused an ~6 log decrease in MSSA CFU/mL (trypsin) (*p* < 0.0001) for both sonicated and unsonicated samples. Sonication significantly increased AMK activity (*p* = 0.0006) in TSB, albeit the actual decrease in CFU/mL (trypsin) was small (~0.5 log). In pSynF, AMK treatment decreased total CFU by at most 1.5 log, whether the sample was sonicated or sonicated+diluted. In human SynF, AMK treatment caused an ~1 log decrease (*p* = 0.0053) in CFU/ml (trypsin). With sonication, AMK in SynF displayed a 5.5 log decrease in CFU/mL(trypsin) (*p* < 0.0001). When human SynF was diluted to 20%, 30 μg/mL AMK caused an ~4 log decrease in CFU/ml(trypsin) (*p* < 0.0001). Sonication of 20% SynF containing 30 μg/mL AMK decreased recovered MSSA counts by ~7.5 logs (*p* ≤ 0.0001). The same trends were observed with *S. epidermidis* in the presence of AMK using TSB and SynF (Supplementary Fig. S1); effects of ultrasound on vancomycin (VAN) activity against MSSA was only observed in 100% SynF (Supplementary Fig. S2). Overall, the effects of diluting the SynF cultures followed by application of ultrasound markedly increased antibiotic sensitivity of bacteria.

### Dilution and clinical ultrasound increase metabolism and antibiotic susceptibility

**Fig. 4:**
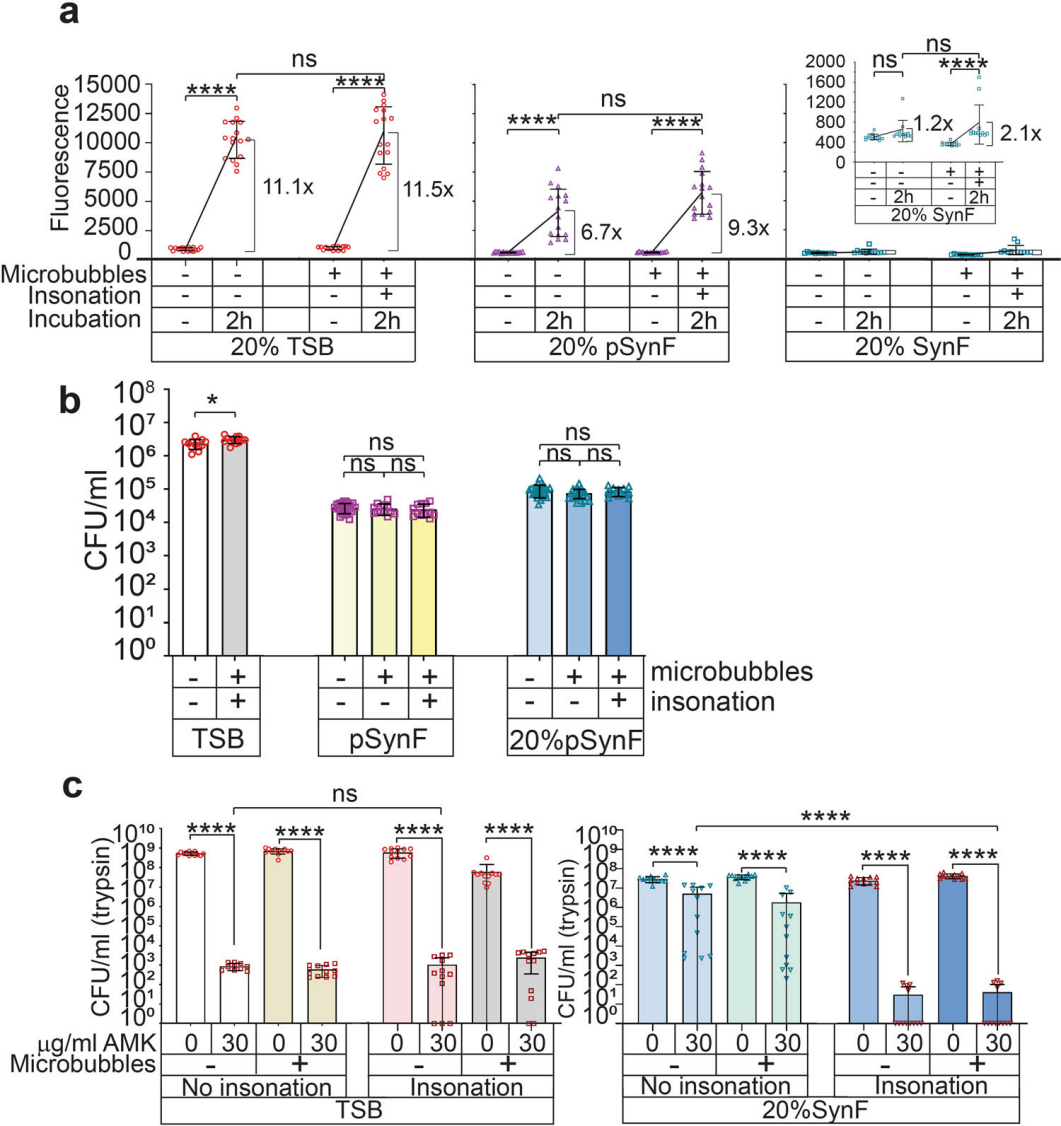
Dilution and clinical ultrasound to disperse aggregates. **a** Metabolic activity, as indicated by Alamar blue fluorescence, of *S. aureus* in 20% TSB, 20% pSynF, or 20% SynF with or without UTMD. Both 20% pSynF and 20% SynF showed an approximate doubling of metabolic activity after UTMD and 2 h incubation. **b** Dispersion measured as change in CFUs after application of clinical ultrasound, with or without microbubbles, to undiluted or diluted pSynF containing *S. aureus* aggregates. No changes in dispersal were measured. **c** AMK sensitivity of MSSA in TSB or 20% SynF as a function of microbubble addition and UTMD. Whereas TSB had 5–6 log decreases in recovered counts, independent of treatments, AMK sensitivity in 20% SynF cultures increased from an ~1 log decrease to a 6-log decrease with the application of ultrasound. Data are presented as mean ± standard deviation. For each bar **a** *n* = 12, **b**, *n* = 12, **c** *n* = 10 (TSB, no insonation, no microbubbles, AMK) or 11 (20% SynF, no insonation, no microbubbles, no AMK) or 12. Individual data points are shown as open, colored symbols. Normality checked using Kolmogorov–Smirnov test. Comparisons done using (**a**, **b** (for pSynF, 20% pSynF)) Kruskal–Wallis test for multiple comparisons or (**b** (for TSB), **c**) Mann-Whitney tests for paired comparison on the basis of independent groups. ns, *P* > 0.05; *P < 0.05; ***P < 0.001; ****P < 0.0001.

Bath sonication applies forces greater than that achieved with the readily translatable clinical ultrasound, and we reasoned that we could augment the mechanical forces achieved with clinical ultrasound through use of UTMD. As with bath sonication, UTMD did not alter MSSA metabolism when measured in 20% TSB (Fig. 4a). Similarly, pSynF with MSSA aggregates diluted to 20% showed no significant increase in metabolic rate after UTMD. The metabolic rate measured in human SynF was low, and UTMD caused an ~2× increase. In terms of MSSA aggregate dispersal, UTMD caused a slight increase in CFU/mL in TSB (*p* = 0.0196). However, no increase in CFU/mL was measured in pSynF, independent of UTMD or dilution (Fig. 4b).

We then asked the effects of the insonation ± microbubbles on antibiotic activity. The presence of 30 µg/mL of AMK reduced MSSA CFU/mL (trypsin) in TSB by 5–6 logs (*p* < 0.0001), independent of the presence of microbubbles or application of insonation (Fig. 4c). On their own in 20% human SynF, the addition of 100 μL/mL microbubbles had no apparent toxicity and 30 μg/mL AMK caused a 1–2 log decrease in CFU/mL (trypsin). Application of clinical ultrasound to MSSA in 20% human SynF containing 30 μg/mL AMK resulted in a 5–6 log decrease in CFU/mL; the use of UTMD did not increase this effect.

### UTMD increases AMK activity in a porcine model of septic arthritis

**Fig. 5:**
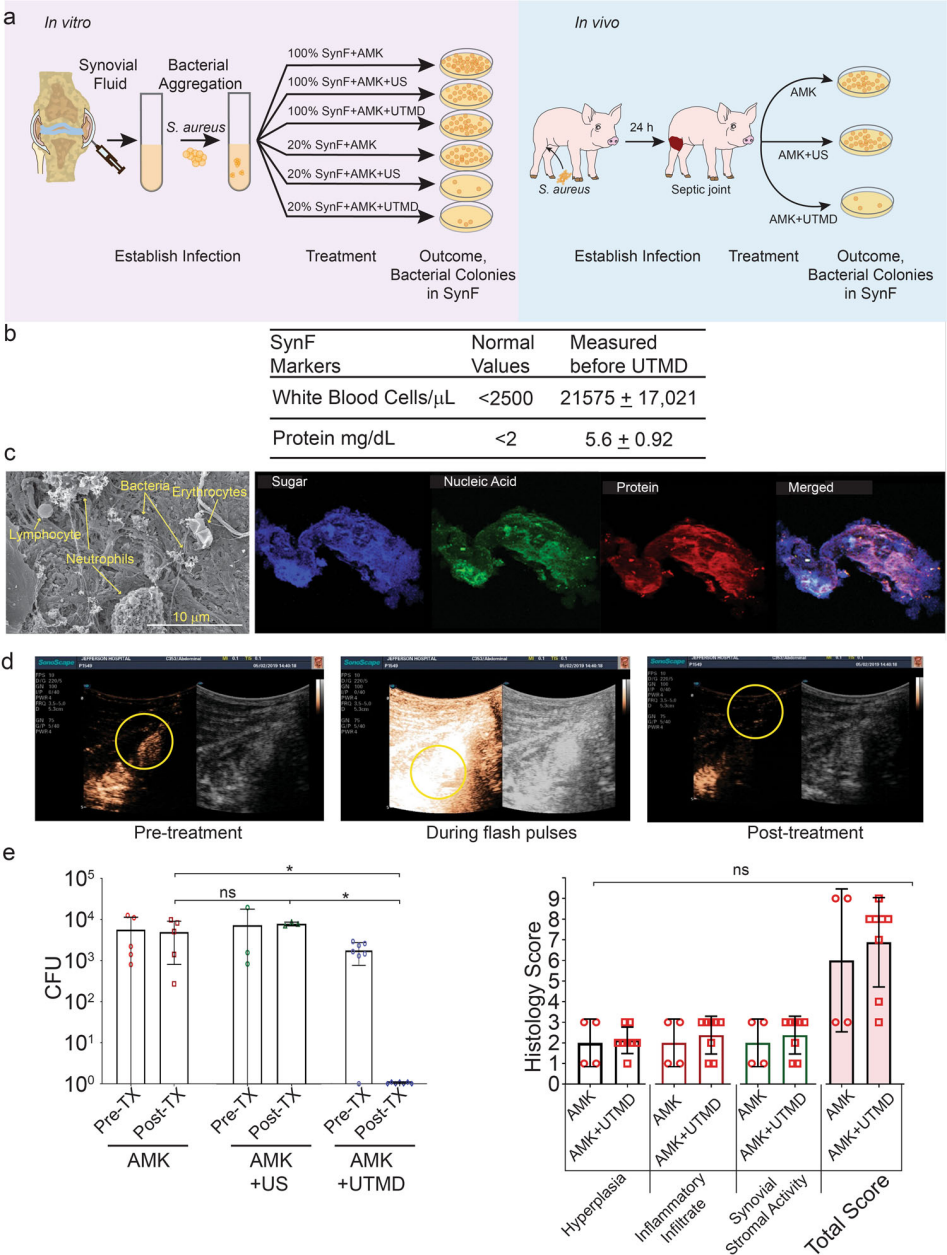
*S. aureus* aggregates and microbubble cavitation in a pig model. **a** (created with BioRender) Cartoon showing comparative in vitro and in vivo treatments. Note that ultrasound+AMK alone is effective in vitro but required AMK + UTMD in vivo. **b** SynF markers before treatment. **c** Representative scanning electron micrograph of 24 h pre-treatment *S. aureus* aggregates in the septic joint. Shown are fibrous *S. aureus* aggregates containing neutrophils, lymphocytes, and erythrocytes. Representative confocal images of an *S. aureus* aggregate from a septic joint. Three-color imaging shows the abundance of sugars, proteins (representative of extracellular matrix), and nucleic acids (representative of extracellular DNA). The merged image shows their relationships. **d** Microbubbles (within yellow circle) in SynF in septic joint. **e** Average recovered CFU in infected joints after treatment with AMK alone (*n* = 5), AMK with insonation (AMK + US; *n* = 3), or AMK with UTMD (n = 7). Comparison of average histological scores for synovium from septic joints treated with AMK alone (*n* = 4) or AMK + UTMD (*n* = 8). Individual data points are shown as open, colored symbols. Values are means ± standard deviation. Comparisons done using Kruskal–Wallis test for multiple comparisons. ns, *P* > 0.05; *P < 0.05; **P < 0.01; ***P < 0.001; ****P < 0.0001.

Finally, we tested the clinical translatability of our in vitro results in a porcine model of septic arthritis. The experimental design tested the in vitro conclusions in this in vivo model (Fig. 5a), where the dilution that was necessary for vitro was hypothesized to be achieved by the addition of AMK and microbubbles. At 24 h post-inoculation with MSSA (*S aureus* ATCC 25923), all animals were visibly lame and infected joints were hot and painful to palpation. SynF samples obtained from the septic joints of anesthetized animals showed elevated nucleated cell counts and protein levels (Fig. 5b), consistent with acute septic arthritis. Bacterial aggregates isolated from the SynF were embedded in a matrix decorated with erythrocytes, neutrophils, and lymphocytes, as visualized by SEM (Fig. 5c). MSSA aggregates were rich in host components, as evidenced by staining for carbohydrates, proteins, and nucleic acids (Fig. 5c). After injection of 100 μL microbubbles + 1 mL of AMK (250 mg) into the femorotibial joint, contrast-enhanced harmonic imaging was able to successfully localize (Fig. 5d) and burst the microbubbles (Fig. 5d) inside the femorotibial joint using higher intensity flash pulses to generate microbubble cavitation. Post-UTMD showed depletion of microbubbles (Fig. 5d). Six to eight hours after treatment, infected joints treated with AMK only or with ultrasound + AMK without microbubbles showed no significant decrease in bacterial burden compared to pre-treatment (Fig. 5e). After UTMD + AMK treatment of infected joints, bacterial counts were not detected in any retrieved SynF (pre-treatment vs. treatment, *p* = 0.0099). Furthermore, AMK + UTMD resulted in a significant decrease in CFU compared to either AMK alone (*p* = 0.0204) or AMK + insonation (*p* = 0.0187); no significant difference was observed between AMK and AMK + US treatments. Necropsy of all joints at time of sacrifice presented with marked hyperemic synovial thickening, associated with fibrinous strands. When synovial tissues were scored (blinded) using the Krenn synovitis score (grade 0–9)^32^ based on tissue hyperplasia (0–3), inflammatory infiltrate (0–3), and synovial stromal activity (0–3), total scores associated with infected synovia treated with AMK + UTMD were not significantly different than the scores for AMK alone (Fig. 5e) for either the individual measures or total scores (Individual scores for each animal, Supplemental Table S5).

## Discussion

Treatment of the septic joint includes debridement of the pannus which is laden with fibrinous bacterial aggregates^33^ and further acts as a barrier to antibiotic access. The presence of bacterial aggregates is apparent in the lavage fluid and corresponds to in vitro aggregates. Thus, joint lavage and, when required, debridement are critical, as reflected in the common treatments of septic arthritis. At the end of these treatments, systemic antibiotics are initiated, with antibiotics placed into the saline-lavaged joint in a subset of cases (often containing implants)^34–36^. This placement of antibiotics after lavage creates the conditions that we have described for the in vitro studies, i.e., bacterial aggregates that have been put into a dilute environment rich in antimicrobials. Because ultrasound application has been used to amplify the effects of antibiotics against bacterial biofilms in rabbits and horses^22,24,37^, we reasoned that the microbial aggregates in the joint might respond to this mechanical perturbation. A major goal of our investigation was to determine if UTMD could restore antibiotic activity in SynF, both in vitro and in vivo.

Most acute septic joint infections are characterized by large numbers of aggregated bacteria. These mucinous aggregates are bacterial biofilms that are rich in serum proteins and decorated with host cells^8,38^. In this manuscript, we showed that SynF MSSA aggregates displayed a marked reduction in antibiotic susceptibility and reduced metabolic activity, consistent with acquisition of a “persister” phenotype. Additionally, antibiotic tolerance was largest at higher bacteria numbers. This inoculum effect has been described for antibiotics in the penicillin, cephalosporin, and fluoroquinolone families and is attributed to decreased antibiotic-bacterial target affinity, antibiotic quenching, the inverse correlation between bacterial density and metabolism, and the production of antibiotic degradative enzymes^39–41^. For AMK, the inoculum effect is attributable to irreversible binding between AMK and the 30 S ribosomal subunit^42^. While in vitro results often don’t predict in vivo results, data showing decreased antibiotic sensitivity at higher bacterial numbers is consistent with clinical experience of improved outcome when treatment is initiated early in the time course of septic arthritis^43,44^.

To enhance antibiotic sensitivity in clinically retrieved SynF and a synthetic pSynF and ultimately treat septic arthritis, we turned to ultrasound-mediated methods. Effects of ultrasound on bacterial biofilm phenotype have been shown to depend on the ultrasound parameters, strains studied, and experimental conditions^45–50^. We initially turned to a bath sonicator as a means to screen through different treatment possibilities. Once we had determined that dilution was required to increase AMK efficacy against bacteria within SynF, an important finding which would be replicated by the joint lavage that is common in treatment of septic joints, we then investigated conditions that would allow similar effects to be realized using clinically-relevant ultrasound probes and energies. Even then, we were merely assured that an insonation effect was possible. Another transition was then required to the complex, constrained space within the in vivo diarthrodial joint model. As we show, each one of these steps required changes with the final testing in the porcine model requiring the additional energy associated with the rupture of ultrasound contrast agents. In vitro, using MSSA aggregates, water bath sonication only caused minimal bacterial dispersal, but restored antibiotic susceptibility to some extent in pSynF and to a greater extent in human SynF. This increase in antibiotic susceptibility could be due to ultrasonic pressure waves subjecting the aggregates to persistent cycles of expansion and contraction. At a microscopic level, this bioacoustic effect would enhance transportation of antibiotics across the bacterial cell membrane^51^^,^^52^. In vitro, these effects have proven to be frequency and energy-dependent and to some extent, we saw this when we looked at the difference in MSSA responsiveness to AMK vs. vancomycin after sonication. We did not pursue this further as both antibiotics showed US-enhanced antibiotic activity and while VAN sensitivity was not increased with dilution, it remained enhanced by the application of US. Importantly, in this work, we show that the media environment modulates effects of ultrasound on bacteria and that this knowledge can be roughly translated to an in vivo situation.

Because lavage removes much of the infected SynF during treatment of septic arthritis, remaining bacteria within the infected joint are exposed to a SynF diluted by antibiotics and saline. Therefore, we mimicked this environment by dilution of aggregate-containing SynF and pSynF. Dilution of the SynF after aggregation in concert with water bath sonication increased MSSA antibiotic susceptibility and metabolism, possibly by allowing nutrient-richer media to infiltrate into the nutrient-depleted aggregate interior^50^. In our experiments using the inflamed pig joint, intraarticular injection of antibiotics and microbubbles diluted the SynF, even in the absence of lavage. In the pig joint, however, ultrasound alone was insufficient and required UTMD to restore antimicrobial efficacy.

Microbubble rupture caused by UTMD subjects the aggregates to shockwaves to transiently permeabilize the cell membrane, increase antibiotic transport, increase biofilm extracellular matrix porosity, and decrease biofilm thickness^21,53^. UTMD is minimally invasive and used for other clinical therapeutic applications^54–56^, including permeabilizing tumors for drug delivery^57^, targeting gene delivery to specific tissues^58^, and facilitating lysis of fibrin clots^59^. Together, these mechanisms are all appropriate for the observed in vitro facilitation of antibiotic activity and bacterial metabolism. These effects were most marked after dilution of SynF, even after aggregation had occurred. This suggests a modality that could be coupled with post-arthroscopic lavage to improve outcomes of treatments for septic joints.

Finally, we tested the ultrasound/antibiotic combination in an animal model of septic arthritis. We chose the pig as we had recently presented the horse and pig as a suitable model for SynF investigations, both for in vitro characterization of effects on bacteria, as well as models of septic arthritis^10^. The pig anatomy, physiology^60^, and innate immune system^61,62^ are very similar to those of humans, and the microvasculature^62^ and microarchitecture^63,64^ of porcine bone closely resembles that of human bone. 24 h after injection of MSSA, the injected joint showed clinical signs of septic arthritis and the SynF contained aggregates that showed dense bacterial clustering in a proteinaceous, mucinous matrix, surrounded by red and white blood cells.

Our in vivo trials with this porcine model of septic arthritis showed no effect of insonation on antibiotic activity in the absence of microbubbles, unlike our in vitro trials where ultrasound was sufficient to increase AMK activity against MSSA aggregates. UTMD in the presence of AMK was required to result in marked reduction in retrieved CFU from the porcine joint where no bacterial counts could be recovered from SynF harvested 6-8 h after the treatment compared to the >2,000 CFU/ml MSSA present in the SynF prior to treatment. We speculate that this requirement for microbubbles is due to the anatomy of the joint. Unidirectional ultrasound waves would be attenuated/blocked by the physical barriers associated with the anatomical complexity of the joint. The inertial cavitation of microbubbles provided multidirectional perturbation, allowing for uniform coverage of the joint spaces, and linked the in vitro findings with these promising in vivo effects.

There are limitations associated with this study. Our in vitro work initially relied on experiments performed in an artificial SynF (pSynF). However, our findings were reproduced in human SynF, where effects appeared more marked than originally seen in pSynF. As in most in vitro systems, the immune component was absent. Furthermore, the US augmentation of AMK activity could be reproduced in *Staphylococcus epidermidis* after dilution; US augmentation of vancomycin activity against MSSA aggregates in SynF did not require this dilution. We do predict that other Staphylococci, including strains of MSSA, MRSA, and *Staphylococcus epidermidis*, will produce similar augmentation of antibiotic activity, although exact antibiotic concentrations and UTMD settings may slightly vary. Finally, our results rely on data from 15 pigs, 7–8 pigs (AMK alone (n = 5), AMK with insonation (AMK + US; n = 3), or AMK with UTMD (n = 7); 7 for bacterial analysis and 8 for histological analysis) of which received UTMD + AMK treatment after development of septic arthritis. In the pigs designated for UTMD + AMK, no bacterial recovery was measured in 7 of 7 joints, 6–8 h after treatment with AMK + UTMD. Furthermore, while the synovium showed clear signs of changes associated with septic arthritis, the destruction of the microbubbles by UTMD did not significantly alter the synovial histology, suggesting that the UTMD itself would not be destructive to the joint. Because our analyses were limited to the 8 h after treatment, the histology reflected the infected environment, where longer times would be necessary to determine if the UTMD-treated animals which displayed greatly reduced bacterial load, would recover synovial integrity. Within those analyses would include an assessment of nucleated cell count and immune cell function. However, these assessments would not be informative, based on our previous work which showed that changes in these parameters required several days after the infection had started to resolve^65^. Thus, additional experiments will be necessary over a longer post treatment survival time to determine if the treatment strategy results in sustained infection clearance and recovery from septic arthritis.

To our knowledge, this is the first report of the application of UTMD to MSSA aggregates for treatment of septic arthritis. Bacterial eradication remains a sought-after treatment; reduction of the bacterial load through local antibiotics allows systemic antibiotics and immune surveillance to control the infection. Our in vitro experiments establish the importance of SynF concentration and ultrasound permeabilization in reduction of bacterial load, especially with regards to the antibiotic-tolerant aggregates that are thought to be a major barrier to successful treatment of septic arthritis. Our in vivo experiments demonstrate the efficacy of this approach and suggest that the combined effects of UTMD and antibiotics may serve as an important adjunct for the treatment of septic arthritides.

## Methods

### Study design

We set out to determine how antibiotic sensitivity could be enhanced in the synovial environment, representative of septic arthritis. The experiments in this study were designed to test the conditions under which ultrasound-triggered microbubble destruction (UTMD) would restore the antibiotic sensitivity of synovial fluid with bacterial aggregates. We tested the ability of antibiotics to eradicate *S. aureus* in different fluids, including human synovial fluid and a model synovial fluid. We tested the ability of insonation and UTMD to increase antibiotic sensitivity both in 100% media and diluted media, as would be encountered in the post-lavage environment of the septic joint (Fig. S1). We then tested the successful treatments in a porcine model of septic arthritis using AMK and UTMD. After 6–8 h, synovial fluid was harvested and assessed for bacterial counts. The IACUC of the University of Pennsylvania approved this study designed in accordance with the ARRIVE guidelines. Human synovial fluid that would normally be discarded was obtained from therapeutically necessary joint aspirations or operations. This material, designated as “waste” (no identifiers), was retrieved and designated as “not human research” by the Thomas Jefferson University Office of Human Research Protections, in keeping with the revised Federal Policy for the Protection of Human Subjects (revised Common Rule, 2018).

### Bacterial growth

Methicillin-susceptible *Staphylococcus aureus* (MSSA) ATCC® 25923^TM^ or methicillin-susceptible *Staphylococcus epidermidis* ATCC® 14990^TM^ was grown overnight, 37 °C, 180 rpm, in trypticase soy broth (TSB, Becton-Dickinson, Sparks, MD) from a single colony, and then diluted 1:5 in TSB and cultured for 2 h. Bacteria numbers were determined by comparison to a 0.5 McFarland standard (~10^8^ CFU/mL) or direct plating. Experimental subculture media consisted of TSB, PBS, human SynF, or pSynF^8^. All plating was performed on 3 M^TM^ Petrifilm^TM^ aerobic films (3M, Saint Paul, MN). When 0.25% trypsin, 2.21 mM EDTA (Corning, Corning, NY)dispersion was used, samples were pelleted, washed, resuspended in trypsin, and incubated for 20 min, 180 rpm, 37 °C, re-pelleted, washed, and serial dilution and plating undertaken^8^. In all experiments, the countable range of bacteria on PetriFilms is between 30-1000 and dilutions are used that fall within those ranges.

### Sonication/UTMD conditions

#### Bath sonication

Aggregates were formed in pSynF or human SynF. Bacteria (10^7^ CFU/mL) in TSB, aggregate-containing SynF or pSynF were transferred to 3.81 cm × 6.35 cm sealable LDPE pouches (Global Industrial, B194311), sealed, and sonicated for 0, 10, 20, 30 min using a bath sonicator (Branson 2800 with 40 kHz transducers). Samples without dispersion were serially diluted, plated, and counted. To dilute the medium, volume and/or viscosity was varied by adding PBS and pSynF in different proportions (Supplemental Tables 1–3) after aggregation and prior to sonication. These samples were then transferred to pouches and sonicated in a bath sonicator for 30 min. To detect changes in aggregation, samples were not dispersed and were directly serially diluted, plated, and counted.

#### UTMD

Aggregates were formed in pSynF or SynF, and contents transferred to pouches. For the undiluted samples, 50 μL, and for the diluted samples, 250 μL, of Definity microbubbles were added. For the diluted samples PBS was added to 2.5 mL. Pouches were sealed, submerged in water, and scanned with a Siemens S3000 HELX Evolution ultrasound scanner with a 6C1 curvilinear array (bandwidth 1.5–6.0 MHz), operating in cadence pulse sequence harmonic imagining mode with a transmit frequency of 2.0 MHz, peak negative pressure 2.0 MPa, and a mechanical index of 1.06 for 6 min with bursting flash mode (50 frames at maximum power over 4 s). TSB controls were treated in parallel. Additional controls included no insonation, no microbubbles. Samples without dispersion were serially diluted, plated, and counted.

### Antibiotic susceptibility

MSSA (10^7^ CFU/mL) or *S. epidermidis* in TSB or pSynF/SynF (containing aggregates) were incubated with 0, 30, or 50 μg/mL AMK, VAN, or PBS for controls, 6 h, 37 °C, 125 rpm. At the end of the incubation, bacteria were dispersed with trypsin, and after treatment, diluted and plated, followed by direct counting. *Bath sonication effects*. MSSA in TSB or pSynF (containing aggregates) were treated with 0, 30, or 50 μg/mL AMK, transferred to pouches, bath sonicated for 0, 10, 20, or 30 min, and incubated for 6 h, 37 °C, 125 rpm. Samples were dispersed by trypsin and pipetting/vortexing. Dispersed samples were diluted, plated, and counted.

To determine the effect of the inoculum, MSSA (10^4^–10^7^ CFU/mL) were aggregated in pSynF, treated with 30 μg/mL AMK or PBS, and incubated, 6 h, 37 °C, 125 rpm. TSB was inoculated with MSSA, 30 μg/mL AMK or PBS, and incubated 6 h, 37 °C, 125 rpm. Samples were dispersed by trypsin and pipetting/vortexing. Dispersed samples were diluted, plated, and counted. *Dilution and sonication*: MSSA in TSB or SynF/pSynF containing aggregates were transferred to pouches. Undiluted or diluted samples (Supplemental Table 4) were transferred to pouches, PBS or 30 μg/mL AMK added, and pouches sealed. After bath sonication for 0 or 30 min, pouches were opened and incubated for 6 h, 37 °C, 125 rpm. Samples were dispersed by trypsin and pipetting/vortexing. Dispersed samples were diluted, plated, and counted.

### UTMD

Aggregates were formed in human SynF. Samples were transferred to pouches, 50 µL of 1500 μg/mL AMK or PBS was added, and total volume brought to 2.5 mL with PBS; and 250 µL of Definity microbubbles or PBS were added. TSB controls were prepared in parallel. For treatment with clinical ultrasound, pouches were sealed, submerged, and scanned with a Siemens S3000 HELX Evolution ultrasound scanner with a 6C1 curvi-linear array (bandwidth 1.5–6.0 MHz), operating in cadence pulse sequence with a transmit frequency of 2.0 MHz, peak negative pressure 2.0 MPa, and a mechanical index of 1.06 for 6 min with bursting flash mode (50 frames at maximum power over 4 s). Pouches were opened and incubated, 6 h, 37 °C, 125 rpm. Samples were dispersed by trypsin and pipetting/vortexing. Dispersed samples were diluted, plated, and counted.

### Metabolic activity

MSSA (10^5^ CFU/mL) in TSB or pSynF (containing aggregates) (24-well tissue culture plate) were incubated for 1.5 h, 37 °C, 180 rpm, and 90 μL of each were transferred into a 96-well black clear bottom tissue culture plate (Thermo Scientific, Waltham, MA). Blank wells contained TSB or pSynF alone. To each well, 10 μL of resazurin (alamarBlue^TM^_,_ ThermoFisher) reagent was added, samples incubated, 37 °C, 180 rpm, and fluorescence (λ_ex/em_ = 540/590 nm) measured hourly for 6 h using an Infinite® M1000 plate reader (Tecan, Männedorf, Switzerland). Metabolic activity is expressed as net fluorescence (bacteria well fluorescence – blank well fluorescence).

To measure metabolism after insonation, 200 μL of pSynF samples (including the aggregates) were transferred to pouches. For TSB, 20 μL of 10^8^ CFU/ml MSSA was inoculated into 180 μL TSB directly in pouches. To all pouches, 700 μL PBS and 100 μL alamarBlue^TM^ were added, diluting the samples to 20%. *Bath sonication*. Before sealing, baseline fluorescence (λ_ex/em_ = 540/590 nm) was measured in 100 μL. Following sealing, samples were bath sonicated or incubated at room temperature for 30 min, pouches were opened, incubated for 2 h, 37 °C, 180 rpm, and fluorescence was read in 100 μL. *UTMD*. 100 μL Definity microbubbles were added, and baseline fluorescence was measured. Following sealing, samples were treated using a Siemens S3000 HELX Evolution ultrasound scanner with a 6C1 curvi-linear array (bandwidth 1.5–6.0 MHz), operating in cadence pulse sequence with a transmit frequency of 2.0 MHz, peak negative pressure 2.0 MPa, and a mechanical index of 1.06 for 6 min at room temperature with bursting flash mode (50 frames at maximum power over 4 s). All pouches were opened, incubated for 2 h, 37 °C, 180 rpm, and fluorescence was read in 100 μL.

### Septic arthritis pig model

This study was approved by the University of Pennsylvania IACUC committee and was designed and executed consistent with ARRIVE guidelines. Seventeen Yorkshire pigs (Meck Swine, Lancaster PA) castrated male and female; ~2–3 months old; ~35–40 kg body weight) were acclimated and habituated to veterinary care staff for 7–10 days and housed on natural bedding in pairs. Pigs incompatible to pair housing were single housed with visual, olfactory, auditory and touch contact to herd mates. Pigs either received buprenorphine (0.01 mg/kg IM, SID-QID) and fentanyl (2.5 µg/kg/h transdermal) or buprenorphine (0.01 mg/kg IM) and morphine (0.2 mg/kg IM) pre- and post-arthrocentesis. Prior to induction, animals were sedated in their pens via intramuscular injection with a combination of tranquilizers and analgesics: Dexmedetomidine (0.02-0.04 mg/kg), Midazolam (0.2–0.4 mg/kg), and Butorphanol (0.1–0.2 mg/kg). Animals were then transported from the pens to the surgical suite. After endotracheal intubation, Isoflurane gas anesthetic (1-4%) was administered with an oxygen carrier, and venous access via an auricular vein was established. Following an aseptic preparation of the skin of one femorotibial joint, the joint was inoculated with 1 × 10^6^ CFU/mL MSSA (ATCC 25923) in 1 mL saline with a sterile gloved hand using a 3.81 cm 20-gauge hypodermic needle. After the procedure animals were recovered from general anesthesia under close veterinary supervision until standing and ambulating. Then they were returned to their pen and housed in pairs or single as described above under a natural light-dark cycle. Animals were examined by a veterinarian at 12 and 24 h following joint injections for signs of joint sepsis, systemic health, and gait asymmetry. After 24 h following joint injection, animals were again premedicated and placed under orotracheal inhalant anesthesia as described above. Following an aseptic prep of the skin of the infected joint, ~200–500 µL SynF were collected from the infected joints for microbial plate and nucleated cell counts and total protein content. Then, five pigs received intraarticular AMK (1 mL of AMK (250 mg)) only into the infected femorotibial joint and three pigs received intraarticular AMK (1 mL of AMK (250 mg)) combined with ultrasound treatment using the identical protocol as described below. The remaining nine pigs received intraarticular injection of 100 µL of Definity microbubbles and 1 mL of AMK (250 mg). The microbubble-containing joints were insonated using a S9 Pro scanner (Sonoscape, Medical Corp., Shenzhen, Guangdong, China) fitted with a curvilinear C353 probe (bandwidth: 2.0–6.8 MHz). Briefly, the access window to the femorotibial joint in this study was via a dorso-medial and dorso-lateral approach using a curvilinear probe. The caudal (posterior) joint space in pigs is very narrow and does not allow for targeted scanning. The joint was taken through multiple range of motion flexion-extension during the UTMD procedure to allow for redistribution of joint fluid. Pulse inversion harmonic imaging with flash replenishment (MI < 0.15) and 4 s destructive pulses (MI > 0.6) was used to burst the microbubbles. Blood was collected pre-inoculation, pre-treatment, and post treatment and submitted for CBC/chemistry analysis. Then animals were recovered from general anesthesia. After 6-8 h post treatment, animals were euthanized with an overdose of Pentobarbital 1 ml/5 kg) according to the guidelines set forth by the current AVMA Panel on Euthanasia and SynF samples were immediately collected from all infected joints for cytological analysis and bacterial quantification. To determine bacterial load, all samples were treated with proteinase K (200 µg/mL) for 10 min and serial threefold dilutions were cultured from all samples and processed for Petrifilm assays to assess bacterial titers (CFU/mL). Then target tissues (synovium and samples of articular cartilage) were harvested and processed for histology.

### Histological assessments

Tissue staining of 10% neutral buffered formalin fixed synovium samples was performed using Brown and Hopps to visualize Gram (+) and (−) bacteria and H&E for tissue ultrastructure. All sections were deparaffinized with xylene and rehydrated in a graded series of ethanol from 100 – 70% (2 × 2 min each) before moving to distilled water. For Brown and Hopps, slides were transferred to 1% Crystal Violet Stain (2 min.) rinsed in DI water to Gram’s Iodine (5 min.), and then rinsed in DI water. Excess water was blotted before decolorizing one slide at a time in acetone until the liquid ran clear, followed by rinsing in running tap water. Basic Fuchsin Stain, 0.25% was applied then rinsed in running tap water. Gallego Solution (5 min.) was used to differentiate then rinsed in running tap water. The slides were then dipped in acetone for 1–2 quick dips, Picric Acid-Acetone 0.05% for 3–10 dips, and Acetone-Xylene 1:1 for 5 dips. Slides were cleared in three changes of xylene, 10 dips each, and then mounted using permount. Sections were visualized by microscopy and scored based on Krenn’s synovial scale^32^ by a microscopist blinded to treatment.

### Statistics and reproducibility

Statistical analyses were performed using Prism statistics. For samples with normal distribution using the Kolmogorov–Smirnov test, ANOVA or Student’s *t* test were used. For those that did not show a normal distribution, either Kruskal–Wallis or Mann–Whitney analyses were used. All measurements were taken from distinct samples.

### Reporting summary

Further information on research design is available in the Nature Portfolio Reporting Summary linked to this article.

## Supplementary information

Supplementary Information

Description of Additional Supplementary Files

Supplementary Data 1

Reporting Summary

## Data Availability

The authors declare that all data supporting the findings of this study are available within the paper and its supplementary information files. Source data can be found in Supplementary Data 1.
