# Supplementary material for: Microbubble cavitation restores Staphylococcus aureus antibiotic susceptibility in vitro and in a septic arthritis model
Source: Commun Biol. 2023 Apr 17;6:425. doi: 10.1038/s42003-023-04752-y (PMC10110534; doi:10.1038/s42003-023-04752-y)
Supplement: Supplementary file 1 — Supplementary Information [file 42003_2023_4752_MOESM1_ESM.pdf]

Table S1. Aggregate dispersal with bath sonication (changing volume, changing protein/viscosity).

	Initial volume		Final volume	Final viscosity
100% TSB	500 µL		500 µL	No change
100% TSB, 30min sonication	500 µL		500 µL	No change
50% TSB, 30min sonication	500 µL	+ 500 µL PBS	1000 µL	No change
20% TSB, 30min sonication	500 µL	+ 2000 µL PBS	2500 µL	No change
100% pSynF	500 µL		500 µL	100%
100% pSynF, 30min sonication	500 µL		500 µL	100%
50% pSynF, 30min sonication	500 µL	+ 500 µL PBS	1000 µL	50%
20% pSynF, 30min sonication	500 µL	+ 2000 µL PBS	2500 µL	20%

Table S2. Aggregate dispersal: Changing volume, keeping viscosity constant.

	Initial volume		Final volume	Final viscosity
100% TSB	500 µL		500 µL	No change
100% TSB, 30min sonication	500 µL		500 µL	No change
50% TSB, 30min sonication	500 µL	+ 500 µL TSB	1000 µL	No change
20% TSB, 30min sonication	500 µL	+2000 µL TSB	2500 µL	No change
100% pSynF	500 µL		500 µL	100%
100% pSynF, 30min sonication	500 µL		500 µL	100%
50% pSynF, 30min sonication	500 µL	+500 µL pSynF	1000 µL	100%
20% pSynF, 30min sonication	500 µL	+2000 µL pSynF	2500 µL	100%

Table S3. Aggregate dispersal: Keeping volume constant, changing viscosity.

	Initial volume		Final volume	Final viscosity
100% TSB	500 µL		500 µL	No change
100% TSB, 30min sonication	500 µL	+ 2000 µL TSB	2500 µL	No change
50% TSB, 30min sonication	500 µL	+ 750 µL TSB<br>+ 1250 µL PBS	2500 µL	No change
20% TSB, 30min sonication	500 µL	+ 2000 µL PBS	2500 µL	No change
100% pSynF	500 µL		500 µL	100%
100% pSynF, 30min sonication	500 µL	+ 2000 µL pSynF	2500 µL	100%
50% pSynF, 30min sonication	500 µL	+ 1250 µL PBS<br>+ 750 µL pSynF	2500 µL	50%
20% pSynF, 30min sonication	500 µL	+ 2000 µL PBS	2500 µL	20%

Table S4. Keeping volume constant with changing protein/viscosity—effect on viscosity

	Initial volume		Final volume	Final viscosity
20% TSB	500 µL	+ 2000 µL PBS	2500 µL	No change
20% pSynF	500 µL	+ 2000 µL PBS	2500 µL	20%
20% SynF	500 µL	+ 2000 µL PBS	2500 µL	20%

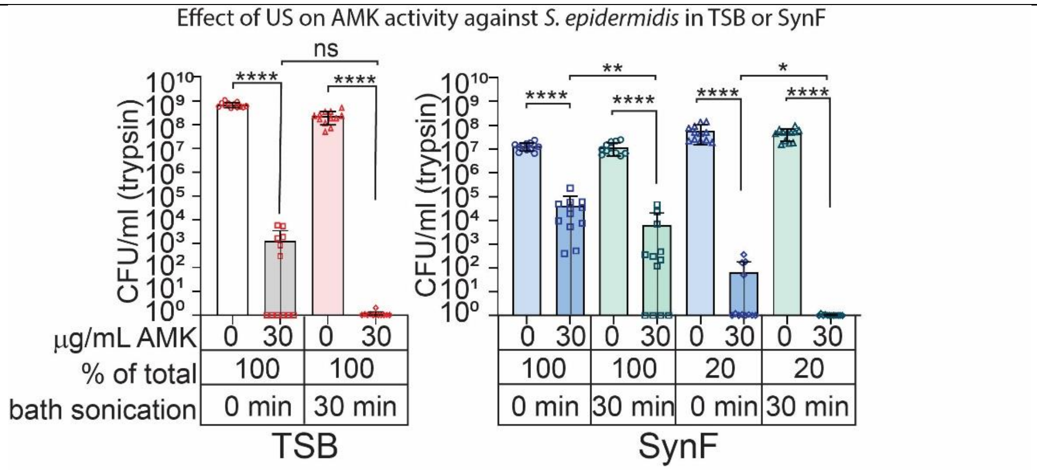

Fig. S1. Dilution and water bath sonication increase *S. epidermidis* AMK sensitivity. The effect of dilution and 30 min sonication on AMK sensitivity of *S. epidermidis* in TSB and SynF. Data are presented as mean ± standard deviation. For each bar n=12. ns=not significant; *$p \leq 0.05$; **$p \leq 0.01$; ****$p \leq 0.0001$.

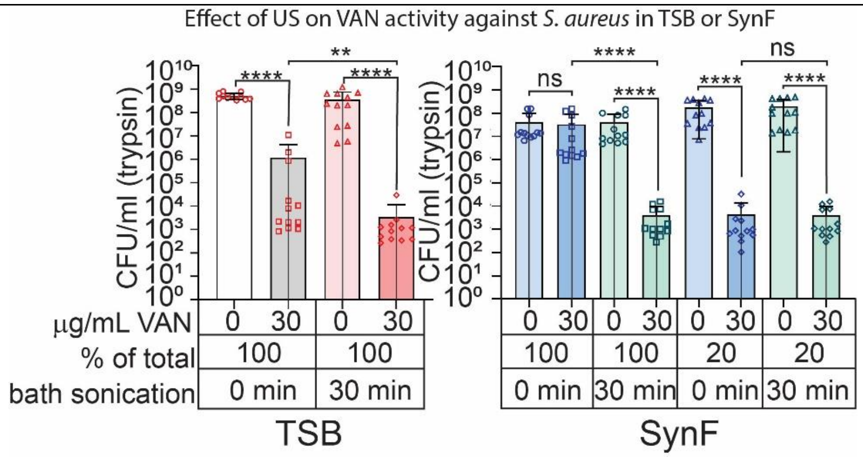

Fig. S2. Dilution and water bath sonication do not alter VAN activity in SynF. The effect of dilution and 30 min sonication on VAN sensitivity of MSSA in TSB and SynF. Data are presented as mean ± standard deviation. For each bar n=12. ns=not significant; *$p \leq 0.05$; **$p \leq 0.01$; ****$p \leq 0.0001$.

Table S5: Scoring data for different parameters for individual joints

	Ear Tag	*Hyperplasia*	*Inflam. Infiltrate*	*Synovial Stromal Activity*	*Total Score*	*Average Score (SD)*	*CFU SynF (pre-TX)*	*CFU SynF (post-TX)*	*Post-TX Bacterial Culture SynF*	*Pre/Post Total Protein SynF (g/dL)*
AMK	605	1	1	1	3	6 (3.46)	11000	8200	+	4.9/4.8
	608	nd	nd	nd	nd		2200	5050	+	4.6/4.5
	2458	3	3	3	9		800	1400	+	4.7/QNS
	2459	1	1	1	3		1400	270	+	4.8/>4.5
	2460	3	3	3	9		12500	9750	+	4.4/>4.5
AMK +MCBL	447	2	3	2	7	6.87 (2.17)	2800	0	-	4.1/4.5
	448	2	3	3	8		2500	0	-	4.2/4.8
	449	3	3	3	9		0	0	-	4.0/4.5
	550*	nd	nd	nd	nd		not infected			
	606	2	3	3	8		1590	0	-	4.1/4.5
	607	2	3	3	8		1400	0	-	4.2/4.7
	1548	2	1	1	4		2230	0	-	4.5/4.8
	1549**	1	1	1	3		TNTC	0	-	4.2/>4.5
	1550	3	2	3	8		1250	0	-	4.6/4.8
AMK +U/S	2196	nd	nd	nd	nd	nd	800	7390	+	4.8/5.0
	2197	nd	nd	nd	nd		1500	6708	+	4.2/4.6
	1883	nd	nd	nd	nd		18500	8250	+	4.3/4.6

*The absence of counts in #550 for both pre- and post-treatment in the UTMD/AMK cohort was likely due to the inoculum being injected periarticular to the left femorotibial joint. While the joint area presented signs of inflammation, tissue distension, and thus the swine manifested signs of septic arthritis, such as abnormal gait and pain upon palpitation, the joint cavity was not infected. **Swine 1549 was not included in the analysis for bacterial counts as the t=0 counts were too numerous to determine accurately. Notably, even in this instance, bacteria were not detectable after treatment.
