# Supplementary material for: Microbubble cavitation restores Staphylococcus aureus antibiotic susceptibility in vitro and in a septic arthritis model
Source: Commun Biol. 2023 Apr 17;6:425. doi: 10.1038/s42003-023-04752-y (PMC10110534; doi:10.1038/s42003-023-04752-y)
Supplement: Supplementary file 3 — Supplementary Data 1 [file 42003_2023_4752_MOESM3_ESM.pdf]

Fig. 1B

Alamar blue, TSB

Time (h)									
1	156	304	121	227	217	382	108	217	144
2	678	1035	761	1003	993	1084	820	872	735
3	2404	3028	3193	3908	4423	2911	3392	3372	2745
4	9362	11059	12797	15563	17654	10071	13546	13577	10764
5	20583	30319	21308	27309	22281	32884	26031	25490	25014
6	11784	26123	10798	22777	15353	27894	20599	20243	20121

									Time (h)
171	122	213	220	214	279	196	389	71	1
811	788	926	840	810	885	943	1073	734	2
3410	3536	3749	2158	2048	2470	3121	2282	1992	3
13659	14201	14934	6278	5602	9691	13228	5812	4712	4
23532	23254	23032	25231	22142	27503	23392	23235	11773	5
16999	15515	15295	26141	26437	24825	14887	32505	22927	6

Alamar blue, pSynF

Time (h)									
1	61	-22	57	-143	53	80	-15	30	-12
2	79	12	100	-44	103	211	58	59	57
3	179	108	230	88	254	490	138	108	151
4	331	277	473	346	536	933	303	224	323
5	695	682	999	943	1147	1840	656	503	726
6	1336	1321	1873	1941	2175	3149	1288	1013	1472

									Time (h)
-27	-117	-38	78	-36	29	91	-47	-32	1
75	11	30	107	-8	132	106	39	32	2
180	167	103	207	52	334	191	169	130	3
376	430	268	377	179	668	346	385	290	4
814	1050	616	773	466	1379	671	890	666	5
1588	2048	1273	1470	995	2417	1219	1756	1326	6

Fig. 1C

Concentration Breakpoint:

pSynF, CFU/mL (Trypsin)

10^4	10^4 + AMK	10^5	10^5+ AMK	10^6	10^6+ AMK	10^7	10^7+ AMK
480000	1	3.1e+007	1	1.44e+008	30	1.42e+009	760
590000	80	5.7e+007	1	1.6e+008	500	1.8e+009	1440
480000	680	1.3e+007	1	1.62e+008	140	1.64e+009	1920
260000	930	2.2e+007	70	1.42e+008	10300	1.2e+009	880
250000	1	2.6e+007	540	1.76e+008	3600	1.36e+009	1020
450000	2550	1.6e+007	8700	1.44e+008	18000	1.26e+009	720
3.2e+007	1	2.32e+008	1	5.9e+008	1	1.18e+009	1280
3.4e+007	1	2.88e+008	170	5.7e+008	1	1.7e+009	1760
2.4e+007	1	2.16e+008	1	6.2e+008	5000	1.3e+009	1220
3.7e+007	1	2.64e+008	460	8.4e+008	10600	1.56e+009	1880
2.1e+007	280	2.76e+008	1	6.3e+008	2960000	1.2e+009	1300
1.8e+007	1040	2.4e+008	5100	8.6e+008	1800000	1.18e+009	1340
7260000	1	7e+007	1	5.4e+008	1	1.6e+009	1700
4320000	1	3.45e+008	1	4.4e+008	1	1.65e+009	570
2950000	1	7.5e+007	1	8.3e+008	1	1.45e+009	1000
3150000	1	1.85e+008	510	7.1e+008	1	2e+009	2200
1900000	70	7.6e+007	740	4.3e+008	110	1.74e+009	2500
2.6e+007	110	7.2e+007	160	4.7e+008	1520	1.86e+009	2350

Test for normal distribution

Kolmogorov-Smirnov test	10^4	10^4+AMK	10^5	10^5+AMK	10^6	10^6+AMK	10^7	10^7+AMK
KS distance	0.2677	0.3486	0.2664	0.4194	0.2037	0.5121	0.1864	0.2646
P value	0.0014	<0.0001	0.0015	<0.0001	0.0467	<0.0001	0.0985	0.0017
Passed normality test (alpha=0.05)?	No	No	No	No	No	No	Yes	No
P value summary	**	****	**	****	*	****	ns	**
Number of values	18	18	18	18	18	18	18	18

Mann-Whitney Test for Different Pairs

Table Analyzed	SA dose pSynF
Column B	10^4+AMK
vs.	vs.
Column A	10^4
Mann Whitney test	
P value	<0.0001
Exact or approximate P value?	Exact
P value summary	****
Significantly different (P < 0.05)?	Yes
One- or two-tailed P value?	Two-tailed
Sum of ranks in column A,B	495 , 171
Mann-Whitney U	0
Difference between medians	
Median of column A	3735000, n=18
Median of column B	1.000, n=18
Difference: Actual	-3734999
Difference: Hodges-Lehmann	-3733725

Table Analyzed	SA dose pSynF
Column D	10^5+AMK
vs.	vs.
Column C	10^5
Mann Whitney test	
P value	<0.0001
Exact or approximate P value?	Exact
P value summary	****
Significantly different (P < 0.05)?	Yes
One- or two-tailed P value?	Two-tailed
Sum of ranks in column C,D	495 , 171
Mann-Whitney U	0
Difference between medians	
Median of column C	75500000, n=18
Median of column D	35.50, n=18
Difference: Actual	-75499965
Difference: Hodges-Lehmann	-75495650

Table Analyzed	SA dose pSynF
Column F	10^6+AMK
vs.	vs.
Column E	10^6
Mann Whitney test	
P value	<0.0001
Exact or approximate P value?	Exact
P value summary	****
Significantly different (P < 0.05)?	Yes
One- or two-tailed P value?	Two-tailed
Sum of ranks in column E,F	495 , 171
Mann-Whitney U	0
Difference between medians	
Median of column E	505000000, n=18
Median of column F	320.0, n=18
Difference: Actual	-504999680
Difference: Hodges-Lehmann	-503520000

Table Analyzed	SA dose pSynF
Column H	10^7+AMK
vs.	vs.
Column G	10^7
Mann Whitney test	
P value	<0.0001
Exact or approximate P value?	Exact
P value summary	****
Significantly different (P < 0.05)?	Yes
One- or two-tailed P value?	Two-tailed
Sum of ranks in column G,H	495 , 171
Mann-Whitney U	0
Difference between medians	
Median of column G	1040000000, n=18
Median of column H	7660000, n=18
Difference: Actual	-1032340000
Difference: Hodges-Lehmann	-1012000000

TSB, CFU/mL (Trypsin)

10^4	10^4 + AMK	10^5	10^5+ AMK	10^6	10^6+ AMK
920000	1	2.2e+007	1	4.9e+008	1
1140000	1	2.6e+007	1	3e+008	1
1140000	1	5e+007	1	4.7e+008	1
1040000	1	4.6e+007	1	3.9e+008	1
1080000	1.1	3.7e+007	1	3.6e+008	100
880000	1	1.8e+007	1	4.1e+008	80
190000	1	2040000	1	1.6e+008	1
180000	1	2360000	1.1	1.6e+008	1
280000	1	3700000	1	1.7e+008	1
300000	1	4150000	1	4.6e+007	1
480000	1	3780000	1	3.25e+007	40
260000	1	2560000	1	4e+007	70
410000	1	2440000	1	1.7e+008	1
440000	1	2450000	1	2e+008	1
410000	1	3600000	1	1.7e+008	1
310000	1	1900000	1	1.5e+008	1
340000	1	2800000	1	7e+007	70
370000	1	3100000	1	6e+007	60

Test for normal distribution

Kolmogorov-Smirnov test	10^4	10^4+AMK	10^5	10^5+AMK	10^6	10^6+AMK	10^7	10^7+AMK
KS distance	0.2614	0.5376	0.3725	0.5376	0.2247	0.4100	0.1204	0.1214
P value	0.0021	<0.0001	<0.0001	<0.0001	0.0168	<0.0001	>0.1000	>0.1000
Passed normality test (alpha=0.05)?	No	No	No	No	No	No	Yes	Yes
P value summary	**	****	****	****	*	****	ns	ns
Number of values	18	18	18	18	18	18	18	18

Mann-Whitney Test forDifferent Pairs

Table Analyzed	TSB concentration SA breakpoint
Column B	10^4 + AMK
vs.	vs.
Column A	10^4
Mann Whitney test	
P value	<0.0001
Exact or approximate P value?	Exact
P value summary	****
Significantly different (P < 0.05)?	Yes
One- or two-tailed P value?	Two-tailed
Sum of ranks in column A,B	495 , 171
Mann-Whitney U	0
Difference between medians	
Median of column A	410000, n=18
Median of column B	1.000, n=18
Difference: Actual	-409999
Difference: Hodges-Lehmann	-409999

Table Analyzed	TSB concentration SA breakpoint
Column D	10^5+ AMK
vs.	vs.
Column C	10^5
Mann Whitney test	
P value	<0.0001

Exact or approximate P value?	Exact
P value summary	****
Significantly different (P < 0.05)?	Yes
One- or two-tailed P value?	Two-tailed
Sum of ranks in column C,D	495 , 171
Mann-Whitney U	0
Difference between medians	
Median of column C	3650000, n=18
Median of column D	1.000, n=18
Difference: Actual	-3649999
Difference: Hodges-Lehmann	-3649999

Table Analyzed	TSB concentration SA breakpoint
Column H	10^{7}+ AMK
vs.	vs.
Column G	10^{7}
Mann Whitney test	
P value	<0.0001
Exact or approximate P value?	Exact
P value summary	****
Significantly different (P < 0.05)?	Yes
One- or two-tailed P value?	Two-tailed
Sum of ranks in column G,H	495 , 171
Mann-Whitney U	0
Difference between medians	
Median of column G	1505000000, n=18
Median of column H	1320, n=18
Difference: Actual	-1504998680
Difference: Hodges-Lehmann	-1504998465

Table Analyzed	TSB concentration SA breakpoint
Column F	10^{6}+ AMK
vs.	vs.
Column E	10^{6}
Mann Whitney test	
P value	<0.0001
Exact or approximate P value?	Exact
P value summary	****
Significantly different (P < 0.05)?	Yes
One- or two-tailed P value?	Two-tailed
Sum of ranks in column E,F	495 , 171
Mann-Whitney U	0
Difference between medians	
Median of column E	170000000, n=18
Median of column F	1.000, n=18
Difference: Actual	-169999999
Difference: Hodges-Lehmann	-169999980

Fig. 2B

Sonication time testing dispersal:

CFU/mL (Trypsin)

TSB no soni.	TSB 10 min	TSB 20 min	TSB 30 min	pSynF no soni.	pSynF 10 min	pSynF 20 min	pSynF 30 min
2220000	3060000	2650000	4450000	13100	50400	1060000	190000
2000000	2650000	3250000	4800000	12100	37800	310000	80000
1900000	3300000	2000000	3200000	9600	72600	1000000	390000
1500000	2550000	3000000	5.4e+007	14700	52500	820000	1260000
2820000	2650000	3750000	4950000	9200	26500	14200	1830000
2940000	3150000	3700000	4450000	10600	850000	440000	1160000
3120000	2280000	1560000	3120000	36000	36000	62400	680000
2460000	1450000	2460000	4740000	28800	37200	48000	460000
2100000	2500000	6060000	3050000	28500	26400	110000	310000
1000000	1400000	2340000	3250000	37800	34800	110000	480000
1980000	2100000	2580000	5150000	33600	38400	43800	27500
1400000	2200000	2550000	3250000	25200	53400	53200	27500
1350000	1920000	1600000	3540000	35400	240000	103200	850000
1920000	2280000	1600000	3720000	39600	36000	45000	600000
1860000	1840000	1550000	4500000	28800	38400	71400	44400
1740000	2100000	2760000	4560000	24600	52200	67200	200000
1350000	4140000	1560000	4200000	32400	427800	51100	46400
1080000	4380000	2460000	3150000	32400	58200	300000	65800

Test for normal distribution

Test for normal distribution								
D'Agostino & Pearson test	TSB 0	TSB 10	TSB 20	TSB 30	pSF 0	pSF 10	pSF 20	pSF 30
K2	0.8322	3.346	16.10	47.09	5.420	31.97	9.442	7.885
P value	0.6596	0.1877	0.0003	<0.0001	0.0665	<0.0001	0.0089	0.0194
Passed normality test (alpha=0.05)?	Yes	Yes	No	No	Yes	No	No	No
P value summary	ns	ns	***	****	ns	****	**	*
Anderson-Darling test								
A2*	0.3112	0.4690	0.8798	5.537	0.8031	3.967	2.430	0.9519
P value	0.5206	0.2187	0.0191	<0.0001	0.0302	<0.0001	<0.0001	0.0124
Passed normality test (alpha=0.05)?	Yes	Yes	No	No	No	No	No	No
P value summary	ns	ns	*	****	*	****	****	*
Shapiro-Wilk test								
W	0.9544	0.9303	0.8239	0.3042	0.8857	0.4921	0.6907	0.8436
P value	0.4975	0.1963	0.0034	<0.0001	0.0326	<0.0001	<0.0001	0.0067
Passed normality test (alpha=0.05)?	Yes	Yes	No	No	No	No	No	No
P value summary	ns	ns	**	****	*	****	****	**
Kolmogorov-Smirnov test								
KS distance	0.1212	0.1745	0.1773	0.4994	0.1793	0.4246	0.3373	0.1852
P value	>0.1000	>0.1000	>0.1000	<0.0001	>0.1000	<0.0001	<0.0001	>0.1000
Passed normality test (alpha=0.05)?	Yes	Yes	Yes	No	Yes	No	No	Yes
P value summary	ns	ns	ns	****	ns	****	****	ns
Number of values	18	18	18	18	18	18	18	18

Ordinary one-way ANOVA: two sets so can compare against the 0 value for each set with the individual media

TSB

Table Analyzed	Fig 2B without expt2				
Data sets analyzed	A-D				
ANOVA summary					
F	2.521				
P value	0.0651				
P value summary	ns				
Significant diff. among means (P < 0.05)?	No				
R squared	0.1001				
Brown-Forsythe test					
F (DFn, DFd)	1.051 (3, 68)				
P value	0.3757				
P value summary	ns				
Are SDs significantly different (P < 0.05)?	No				
Bartlett's test					
Bartlett's statistic (corrected)	174.6				
P value	<0.0001				
P value summary	****				
Are SDs significantly different (P < 0.05)?	Yes				
ANOVA table	SS	DF	MS	F (DFn, DFd)	P value
Treatment (between columns)	2.679e+014	3	89285025925926	F (3, 68) = 2.521	P=0.0651
Residual (within columns)	2.408e+015	68	35411122385621		
Total	2.676e+015	71			
Data summary					
Number of treatments (columns)	4				
Number of values (total)	72				

Multiple Comparisons

Number of families	1							
Number of comparisons per family	3							
Alpha	0.05							
Dunnett's multiple comparisons test	Mean Diff.	95.00% CI of diff.	Below threshold?	Summary	Adjusted P Value	A-?		
TSB no soni. vs. TSB 10 min	-622778	-5388896 to 4143341	No	ns	0.9792	B	TSB 10 min	
TSB no soni. vs. TSB 20 min	-705000	-5471118 to 4061118	No	ns	0.9705	C	TSB 20 min	
TSB no soni. vs. TSB 30 min	-4852222	-9618341 to -86104	Yes	*	0.0450	D	TSB 30 min	
Test details	Mean 1	Mean 2	Mean Diff.	SE of diff.	n1	n2	q	DF
TSB no soni. vs. TSB 10 min	1930000	2552778	-622778	1983575	18	18	0.3140	68
TSB no soni. vs. TSB 20 min	1930000	2635000	-705000	1983575	18	18	0.3554	68
TSB no soni. vs. TSB 30 min	1930000	6782222	-4852222	1983575	18	18	2.446	68

pSynF

Table Analyzed	Fig 2B without expt2
Data sets analyzed	F-I
ANOVA summary	
F	6.797
P value	0.0004
P value summary	***

Significant diff. among means (P < 0.05)?	Yes				
R squared	0.2307				
Brown-Forsythe test					
F (DFn, DFd)	6.645 (3, 68)				
P value	0.0005				
P value summary	***				
Are SDs significantly different (P < 0.05)?	Yes				
Bartlett's test					
Bartlett's statistic (corrected)	111.1				
P value	<0.0001				
P value summary	****				
Are SDs significantly different (P < 0.05)?	Yes				
ANOVA table	SS	DF	MS	F (DFn, DFd)	P value
Treatment (between columns)	2141537051528	3	713845683843	F (3, 68) = 6.797	P=0.0004
Residual (within columns)	7141158145000	68	105017031544		
Total	9282695196528	71			
Data summary					
Number of treatments (columns)	4				
Number of values (total)	72				

Multiple Comparisons

Number of families	1
Number of comparisons per family	3
Alpha	0.05

Dunnett's multiple comparisons test	Mean Diff.	95.00% CI of diff.	Below threshold?	Summary	Adjusted P Value	F-?	
pSynF no soni. vs. pSynF 10 min	-95344	-354897 to 164208	No	ns	0.7079	G	pSynF 10 min
pSynF no soni. vs. pSynF 20 min	-236506	-496058 to 23047	No	ns	0.0819	H	pSynF 20 min
pSynF no soni. vs. pSynF 30 min	-458289	-717841 to -198737	Yes	***	0.0002	I	pSynF 30 min

Test details	Mean 1	Mean 2	Mean Diff.	SE of diff.	n1	n2	q	DF
pSynF no soni. vs. pSynF 10 min	25133	120478	-95344	108021	18	18	0.8826	68
pSynF no soni. vs. pSynF 20 min	25133	261639	-236506	108021	18	18	2.189	68
pSynF no soni. vs. pSynF 30 min	25133	483422	-458289	108021	18	18	4.243	68

Fig. 2C

0 min sonication:

pSynF, TSB with AMK; CFU/mL (Trypsin)

TSB			pSynF		
0 µg/ml AMK	30 µg/ml AMK	50 µg/ml AMK	0 µg/ml AMK	30 µg/ml AMK	50 µg/ml AMK
1.47e+009	1	1	5.8e+008	1.14e+007	1
1.13e+009	1	1	5.5e+008	4.68e+007	1
6.1e+008	1.1	1.1	6.7e+008	7.04e+007	2.51e+007
9.2e+008	1	1	3.1e+008	5.32e+007	8500000
4.3e+008	1	1	3.8e+008	5.8e+007	8700000
2.6e+008	0.9	1	8.24e+007	6700000	1.62e+007
1.44e+009	1	0.9	2.5e+008	2.54e+007	2.7e+007
1.5e+009	1	1	3.8e+008	1.72e+007	8200000
1.54e+009	1	1	5.8e+008	2.67e+007	1.85e+007

Test for normal distribution

Test for normal distribution	0 ug/mL AMK	30 ug/ml AMK	50 ug/ml AMK	0 ug/ml AMK	30 ug/ml AMK	50 ug/ml AMK
D'Agostino & Pearson test						
K2	2.255	4.703	4.703	0.4819	1.620	0.8630
P value	0.3239	0.0952	0.0952	0.7859	0.4448	0.6495
Passed normality test (alpha=0.05)?	Yes	Yes	Yes	Yes	Yes	Yes
P value summary	ns	ns	ns	ns	ns	ns
Anderson-Darling test						
A2*	0.4825	1.632	1.632	0.2844	0.2993	0.3158
P value	0.1698	0.0001	0.0001	0.5413	0.5107	0.4706
Passed normality test (alpha=0.05)?	Yes	No	No	Yes	Yes	Yes
P value summary	ns	***	***	ns	ns	ns
Shapiro-Wilk test						
W	0.8743	0.6931	0.6931	0.9457	0.9335	0.9212
P value	0.1366	0.0012	0.0012	0.6431	0.5158	0.4019
Passed normality test (alpha=0.05)?	Yes	No	No	Yes	Yes	Yes
P value summary	ns	**	**	ns	ns	ns
Kolmogorov-Smirnov test						
KS distance	0.2368	0.3889	0.3889	0.1971	0.2003	0.2041
P value	>0.1000	0.0003	0.0003	>0.1000	>0.1000	>0.1000
Passed normality test (alpha=0.05)?	Yes	No	No	Yes	Yes	Yes
P value summary	ns	***	***	ns	ns	ns
Number of values	9	9	9	9	9	9

Kruskal-Wallis Test for non-parametric distributions, multiple Tests

TSB

Number of families	1						
Number of comparisons per family	2						
Alpha	0.05						
Dunn's multiple comparisons test	Mean rank diff.	Significant?	Summary	Adjusted P Value	A-?		
0 µg/ml AMK vs. 30 µg/ml AMK	13.50	Yes	***	0.0002	B	30 µg/ml AMK	
0 µg/ml AMK vs. 50 µg/ml AMK	13.50	Yes	***	0.0002	C	50 µg/ml AMK	
Test details	Mean rank 1	Mean rank 2	Mean rank diff.	n1	n2		Z
0 µg/ml AMK vs. 30 µg/ml AMK	23.00	9.500	13.50	9	9		3.890
0 µg/ml AMK vs. 50 µg/ml AMK	23.00	9.500	13.50	9	9		3.890

<u>pSynF</u>

Number of families	1					
Number of comparisons per family	2					
Alpha	0.05					
Dunn's multiple comparisons test	Mean rank diff.	Significant?	Summary	Adjusted P Value	E-?	
0 **µ**g/ml AMK vs. 30 **µ**g/ml AMK	10.78	Yes	**	0.0079	F	30 **µ**g/ml AMK
0 **µ**g/ml AMK vs. 50 **µ**g/ml AMK	16.22	Yes	****	<0.0001	G	50 **µ**g/ml AMK
Test details	Mean rank 1	Mean rank 2	Mean rank diff.	n1	n2	Z
0 **µ**g/ml AMK vs. 30 **µ**g/ml AMK	23.00	12.22	10.78	9	9	2.882
0 **µ**g/ml AMK vs. 50 **µ**g/ml AMK	23.00	6.778	16.22	9	9	4.338

10 min sonication:

pSynF, TSB with AMK; CFU/mL (Trypsin)

0 µg/ml AMK-TSB	30 µg/ml AMK-TSB	50 µg/ml AMK-TSB	pSynF0 µg/ml AMK	pSynF-30 µg/ml AMK	pSynF-50 µg/ml AMK
1.01e+009	1	1	5.4e+008	5.6e+007	1.03e+007
8.9e+008	1.1	1	7.5e+008	824000	3500000
1.9e+008	1	1.1	1.9e+008	1	1
1.26e+009	1	1	3.8e+008	3.69e+007	1
3.3e+008	1	1	5.5e+008	2.36e+007	6700000
1.41e+007	1	1.1	7e+008	5.96e+007	1500000
1.13e+009	1.1	1	2.2e+008	1.96e+007	1.78e+007
9.7e+008	1	1	3.3e+008	1.75e+007	2400000
1.87e+009	1	1	4.4e+008	2000000	8800000

Test for normal distribution

Test for normal distribution	0 µg/ml AMK-TSB	30 µg/ml AMK-TSB	50 µg/ml AMK-TSB	pSynF0 µg/ml AMK	pSynF-30 µg/ml AMK	pSynF-50 µg/ml AMK
D'Agostino & Pearson test						
K2	0.02479	5.409	5.409	0.6681	1.171	3.231
P value	0.9877	0.0669	0.0669	0.7160	0.5569	0.1988
Passed normality test (alpha=0.05)?	Yes	Yes	Yes	Yes	Yes	Yes
P value summary	ns	ns	ns	ns	ns	ns
Anderson-Darling test						
A2*	0.2996	2.150	2.150	0.1875	0.4062	0.4079
P value	0.5101	<0.0001	<0.0001	0.8652	0.2739	0.2711
Passed normality test (alpha=0.05)?	Yes	No	No	Yes	Yes	Yes
P value summary	ns	****	****	ns	ns	ns
Shapiro-Wilk test						
W	0.9477	0.5358	0.5358	0.9544	0.8883	0.8884
P value	0.6648	<0.0001	<0.0001	0.7380	0.1918	0.1923
Passed normality test (alpha=0.05)?	Yes	No	No	Yes	Yes	Yes
P value summary	ns	****	****	ns	ns	ns
Kolmogorov-Smirnov test						
KS distance	0.1929	0.4706	0.4706	0.1150	0.1737	0.1993
P value	>0.1000	<0.0001	<0.0001	>0.1000	>0.1000	>0.1000
Passed normality test (alpha=0.05)?	Yes	No	No	Yes	Yes	Yes
P value summary	ns	****	****	ns	ns	ns
Number of values	9	9	9	9	9	9

Kruskal-Wallis Test for non-parametric distributions, multiple Tests

TSB

Table Analyzed	10min sonication
Kruskal-Wallis test	
P value	<0.0001
Exact or approximate P value?	Approximate
P value summary	****
Do the medians vary signif. (P < 0.05)?	Yes
Number of groups	3
Kruskal-Wallis statistic	20.23
Data summary	
Number of treatments (columns)	3
Number of values (total)	27

Multiple comparisons

Number of families	1
Number of comparisons per family	2
Alpha	0.05

Dunn's multiple comparisons test	Mean rank diff.	Significant?	Summary	Adjusted P Value	A-?	
0 **µ**g/ml AMK-TSB vs. 30 **µ**g/ml AMK-TSB	13.50	Yes	***	0.0002	B	30 **µ**g/ml AMK-TSB
0 **µ**g/ml AMK-TSB vs. 50 **µ**g/ml AMK-TSB	13.50	Yes	***	0.0002	C	50 **µ**g/ml AMK-TSB
**Test details**	**Mean rank 1**	**Mean rank 2**	**Mean rank diff.**	**n1**	**n2**	**Z**
0 **µ**g/ml AMK-TSB vs. 30 **µ**g/ml AMK-TSB	23.00	9.500	13.50	9	9	3.895
0 **µ**g/ml AMK-TSB vs. 50 **µ**g/ml AMK-TSB	23.00	9.500	13.50	9	9	3.895

pSynF

Table Analyzed	10min sonication
Kruskal-Wallis test	
P value	<0.0001
Exact or approximate P value?	Approximate
P value summary	****
Do the medians vary signif. (P < 0.05)?	Yes
Number of groups	3
Kruskal-Wallis statistic	18.59
Data summary	
Number of treatments (columns)	3
Number of values (total)	27

Multiple Comparisons

Number of families	1
Number of comparisons per family	2
Alpha	0.05

Dunn's multiple comparisons test	Mean rank diff.	Significant ?	Summary	Adjusted P Value	E-?	
pSynF0 **µ**g/ml AMK vs. pSynF-30 **µ**g/ml AMK	11.44	Yes	**	0.0044	F	pSynF-30 **µ**g/ml AMK
pSynF0 **µ**g/ml AMK vs. pSynF-50 **µ**g/ml AMK	15.56	Yes	****	<0.0001	G	pSynF-50 **µ**g/ml AMK
**Test details**	**Mean rank 1**	**Mean rank 2**	**Mean rank diff.**	**n1**	**n2**	**Z**
pSynF0 **µ**g/ml AMK vs. pSynF-30 **µ**g/ml AMK	23.00	11.56	11.44	9	9	3.061
pSynF0 **µ**g/ml AMK vs. pSynF-50 **µ**g/ml AMK	23.00	7.444	15.56	9	9	4.160

20 min sonication:

pSynF, TSB with AMK; CFU/mL (Trypsin)

TSB 0 µg/ml AMK	TSB 30 µg/ml AMK	TSB 50 µg/ml AMK	pSynF 0 µg/ml AMK	pSynF 30 µg/ml AMK	pSynF 50 µg/ml AMK
1.4e+009	1	1	7.4e+008	412000	28000
1.15e+009	1	1	7.4e+008	3.6e+007	19000
5e+008	1.1	1	8.2e+008	8.96e+007	1
3.4e+008	1	1.1	3.5e+008	2.8e+007	880000
3.1e+008	1	1	8.4e+008	5300000	17000
1.3e+008	1	1	6.8e+008	1	1.5e+007
1.87e+009	1	1.1	4.9e+008	1.49e+007	2700000
1.37e+009	1.1	1	2e+008	2.61e+007	9000000
2.1e+009	1	1	5.8e+008	576000	1050000

Test for normal distribution

Test for normal distribution	TSB 0 µg/ml AMK	TSB 30 µg/ml AMK	TSB 50 µg/ml AMK	pSynF 0 µg/ml AMK	pSynF 30 µg/ml AMK	pSynF 50 µg/ml AMK
D'Agostino & Pearson test						
K2	1.911	5.409	5.409	1.425	10.88	8.992
P value	0.3845	0.0669	0.0669	0.4904	0.0043	0.0112
Passed normality test (alpha=0.05)?	Yes	Yes	Yes	Yes	No	No
P value summary	ns	ns	ns	ns	**	*
Anderson-Darling test						
A2*	0.3679	2.150	2.150	0.3539	0.7510	1.308
P value	0.3457	<0.0001	<0.0001	0.3760	0.0315	0.0009
Passed normality test (alpha=0.05)?	Yes	No	No	Yes	No	No
P value summary	ns	****	****	ns	*	***
Shapiro-Wilk test						
W	0.9128	0.5358	0.5358	0.9128	0.7841	0.6842
P value	0.3357	<0.0001	<0.0001	0.3362	0.0134	0.0009
Passed normality test (alpha=0.05)?	Yes	No	No	Yes	No	No
P value summary	ns	****	****	ns	*	***
Kolmogorov-Smirnov test						
KS distance	0.2074	0.4706	0.4706	0.1900	0.2178	0.3237
P value	>0.1000	<0.0001	<0.0001	>0.1000	>0.1000	0.0072
Passed normality test (alpha=0.05)?	Yes	No	No	Yes	Yes	No
P value summary	ns	****	****	ns	ns	**
Number of values	9	9	9	9	9	9

Kruskal-Wallis Test for non-parametric distributions, multiple Tests

TSB

Table Analyzed	20 min sonication
Kruskal-Wallis test	
P value	<0.0001
Exact or approximate P value?	Approximate
P value summary	****
Do the medians vary signif. (P < 0.05)?	Yes
Number of groups	3

Kruskal-Wallis statistic	20.23
Data summary	
Number of treatments (columns)	3
Number of values (total)	27

Multiple comparisons

Number of families	1					
Number of comparisons per family	2					
Alpha	0.05					
Dunn's multiple comparisons test	Mean rank diff.	Significant?	Summary	Adjusted P Value	A-?	
TSB 0 **µ**g/ml AMK vs. TSB 30 **µ**g/ml AMK	13.50	Yes	***	0.0002	B	TSB 30 **µ**g/ml AMK
TSB 0 **µ**g/ml AMK vs. TSB 50 **µ**g/ml AMK	13.50	Yes	***	0.0002	C	TSB 50 **µ**g/ml AMK

Test details	Mean rank 1	Mean rank 2	Mean rank diff.	n1	n2	Z
TSB 0 **µ**g/ml AMK vs. TSB 30 **µ**g/ml AMK	23.00	9.500	13.50	9	9	3.895
TSB 0 **µ**g/ml AMK vs. TSB 50 **µ**g/ml AMK	23.00	9.500	13.50	9	9	3.895

pSynF

Table Analyzed	20 min sonication
Kruskal-Wallis test	
P value	<0.0001
Exact or approximate P value?	Approximate
P value summary	****
Do the medians vary signif. (P < 0.05)?	Yes
Number of groups	3
Kruskal-Wallis statistic	18.64
Data summary	
Number of treatments (columns)	3
Number of values (total)	27

Multiple Comparisons

Number of families	1					
Number of comparisons per family	2					
Alpha	0.05					
Dunn's multiple comparisons test	Mean rank diff.	Significant ?	Summary	Adjusted P Value	E-?	
pSynF 0 **µ**g/ml AMK vs. pSynF 30 **µ**g/ml AMK	11.39	Yes	**	0.0047	F	pSynF 30 **µ**g/ml AMK
pSynF 0 **µ**g/ml AMK vs. pSynF 50 **µ**g/ml AMK	15.61	Yes	****	<0.0001	G	pSynF 50 **µ**g/ml AMK

Test details	Mean rank 1	Mean rank 2	Mean rank diff.	n1	n2	Z
pSynF 0 **µ**g/ml AMK vs. pSynF 30 **µ**g/ml AMK	23.00	11.61	11.39	9	9	3.045
pSynF 0 **µ**g/ml AMK vs. pSynF 50 **µ**g/ml AMK	23.00	7.389	15.61	9	9	4.174

30 min sonication:

pSynF, TSB with AMK; CFU/mL (Trypsin)

TSB 0 µg/ml AMK	TSB 30 µg/ml AMK	TSB 50 µg/ml AMK	pSynF 0 µg/ml AMK	pSynF 30 µg/ml AMK	pSynF 50 µg/ml AMK
4.6e+008	1	1	6.9e+008	1	1
1.76e+009	1	1.1	1.13e+009	1	1
8.7e+008	1	1	8.4e+008	4100000	35000
1.8e+008	1.1	1	3.4e+008	1	3700000
1.9e+008	1	1	3.3e+008	1	8400000
8e+008	1	1.1	8.2e+007	1.11e+007	138000
1.4e+009	1.1	1	4e+008	1.43e+007	8700000
1.92e+009	1	1	5.3e+008	3100000	1.11e+007
1.58e+009	1	1	2.6e+008	347000	672000

Test for normal distribution

Test for normal distribution	TSB 0 µg/ml AMK	TSB 30 µg/ml AMK	TSB 50 µg/ml AMK	pSynF 0 µg/ml AMK	pSynF 30 µg/ml AMK	pSynF 50 µg/ml AMK
D'Agostino & Pearson test						
K2	2.406	5.409	5.409	1.398	4.236	2.440
P value	0.3003	0.0669	0.0669	0.4971	0.1203	0.2952
Passed normality test (alpha=0.05)?	Yes	Yes	Yes	Yes	Yes	Yes
P value summary	ns	ns	ns	ns	ns	ns
Anderson-Darling test						
A2*	0.3187	2.150	2.150	0.2859	1.071	0.8949
P value	0.4627	<0.0001	<0.0001	0.5377	0.0042	0.0127
Passed normality test (alpha=0.05)?	Yes	No	No	Yes	No	No
P value summary	ns	****	****	ns	**	*
Shapiro-Wilk test						
W	0.9138	0.5358	0.5358	0.9465	0.7373	0.7834
P value	0.3436	<0.0001	<0.0001	0.6520	0.0039	0.0132
Passed normality test (alpha=0.05)?	Yes	No	No	Yes	No	No
P value summary	ns	****	****	ns	**	*
Kolmogorov-Smirnov test						
KS distance	0.1602	0.4706	0.4706	0.1896	0.2857	0.2991
P value	>0.1000	<0.0001	<0.0001	>0.1000	0.0329	0.0198
Passed normality test (alpha=0.05)?	Yes	No	No	Yes	No	No
P value summary	ns	****	****	ns	*	*
Number of values	9	9	9	9	9	9

Kruskal-Wallis Test for non-parametric distributions, multiple Tests

TSB

Table Analyzed	30 min sonication
Kruskal-Wallis test	
P value	<0.0001

Exact or approximate P value?	Approximate
P value summary	****
Do the medians vary signif. (P < 0.05)?	Yes
Number of groups	3
Kruskal-Wallis statistic	20.23
Data summary	
Number of treatments (columns)	3
Number of values (total)	27

Multiple comparisons

Number of families	1
Number of comparisons per family	2
Alpha	0.05

Dunn's multiple comparisons test	Mean rank diff.	Significant?	Summary	Adjusted P Value	A-?	
TSB 0 µg/ml AMK vs. TSB 30 µg/ml AMK	13.50	Yes	***	0.0002	B	TSB 30 µg/ml AMK
TSB 0 µg/ml AMK vs. TSB 50 µg/ml AMK	13.50	Yes	***	0.0002	C	TSB 50 µg/ml AMK

Test details	Mean rank 1	Mean rank 2	Mean rank diff.	n1	n2	Z
TSB 0 µg/ml AMK vs. TSB 30 µg/ml AMK	23.00	9.500	13.50	9	9	3.895
TSB 0 µg/ml AMK vs. TSB 50 µg/ml AMK	23.00	9.500	13.50	9	9	3.895

pSynF

Table Analyzed	30 min sonication
Kruskal-Wallis test	
P value	0.0002
Exact or approximate P value?	Approximate
P value summary	***
Do the medians vary signif. (P < 0.05)?	Yes
Number of groups	3
Kruskal-Wallis statistic	17.61
Data summary	
Number of treatments (columns)	3
Number of values (total)	27

Multiple Comparisons

Number of families	1
Number of comparisons per family	2
Alpha	0.05

Dunn's multiple comparisons test	Mean rank diff.	Significant ?	Summary	Adjusted P Value	E-?	
pSynF 0 µg/ml AMK vs. pSynF 30 µg/ml AMK	13.94	Yes	***	0.0004	F	pSynF 30 µg/ml AMK
pSynF 0 µg/ml AMK vs. pSynF 50 µg/ml AMK	13.06	Yes	***	0.0009	G	pSynF 50 µg/ml AMK

Test details	Mean rank 1	Mean rank 2	Mean rank diff.	n1	n2	Z
pSynF 0 µg/ml AMK vs. pSynF 30 µg/ml AMK	23.00	9.056	13.94	9	9	3.747

pSynF 0 **µ**g/ml AMK vs. pSynF 50 **µ**g/ml AMK	23.00	9.944	13.06	9	9	3.509

Fig. 3A

Changing protein, changing volume (straight dilution)

TSB no soni. 100%	TSB 30 min. 100%	TSB 30 min. 50%	TSB 30 min. 20%	pSynF no soni. 100%	pSynF 30 min. 100%	pSynF 30 min. 50%	pSynF 30 min. 20%
2280000	2600000	6480000	1.025e+007	30000	630000	1200000	900000
1360000	2640000	8640000	9250000	25500	420000	2900000	500000
1240000	3000000	7560000	7400000	33000	1050000	1180000	1.56e+007
1800000	3120000	9380000	7800000	21000	840000	25000	350000
1200000	2450000	5800000	8400000	25500	320000	440000	1700000
1320000	2700000	7800000	1.02e+007	20000	590000	560000	300000
2600000	3.4e+007	9360000	6800000	28200	52500	109200	6100000
3180000	2.5e+007	6600000	5800000	36400	52200	61200	1350000
3570000	1.6e+007	5600000	7800000	26400	31800	300000	1.03e+007
2200000	4500000	6200000	4600000	25200	36000	300000	5200000
2880000	2e+007	8160000	7900000	31200	140000	700000	1900000
3120000	2e+007	6400000	9600000	25200	35400	480000	3150000
3000000	2700000	9940000	1.38e+007	13200	140000	2240000	6100000
2880000	2940000	6120000	1.17e+007	22200		680000	4900000
2760000	5280000	7200000	1.2e+007	32900		480000	4400000
3220000	5940000	4100000	1.025e+007	24600	310000	140000	5600000
2400000	4320000	5160000	1.53e+007	31500	130000	400000	4600000
3120000	5760000	4800000	1.44e+007	20400	760000	5400000	6800000

Test for normality

Test for normal distribution	TSB no soni 100%	TSB 30 min. 100%	TSB 30 min. 50%	TSB 30 min. 20%	pSynF no soni. 100%	pSynF 30 min. 100%	pSynF 30 min. 50%	pSynF 30 min. 20%
D'Agostino & Pearson test								
K2	2.767	8.691	0.7633	0.6476	0.5937	2.434	24.74	10.77
P value	0.2507	0.0130	0.6827	0.7234	0.7431	0.2962	<0.0001	0.0046
Passed normality test (alpha=0.05)?	Yes	No	Yes	Yes	Yes	Yes	No	No
P value summary	ns	*	ns	ns	ns	ns	****	**
Anderson-Darling test								
A2*	0.6774	2.242	0.2232	0.2676	0.2461	0.8373	2.204	0.7142
P value	0.0638	<0.0001	0.7951	0.6425	0.7179	0.0238	<0.0001	0.0512
Passed normality test (alpha=0.05)?	Yes	No	Yes	Yes	Yes	No	No	Yes
P value summary	ns	****	ns	ns	ns	*	****	ns
Shapiro-Wilk test								
W	0.9022	0.7177	0.9704	0.9673	0.9729	0.8634	0.6710	0.8636
P value	0.0628	0.0001	0.8052	0.7452	0.8506	0.0216	<0.0001	0.0140
Passed normality test (alpha=0.05)?	Yes	No	Yes	Yes	Yes	No	No	No
P value summary	ns	***	ns	ns	ns	*	****	*
Kolmogorov-Smirnov test								
KS distance	0.1556	0.3497	0.1414	0.1382	0.1091	0.2326	0.3043	0.1678
P value	>0.1000	<0.0001	>0.1000	>0.1000	>0.1000	0.0207	0.0001	>0.1000
Passed normality test (alpha=0.05)?	Yes	No	Yes	Yes	Yes	No	No	Yes
P value summary	ns	****	ns	ns	ns	*	***	ns
Number of values	18	18	18	18	18	16	18	18

Kruskal-Wallis as not parametric

TSB

Table Analyzed	Changing protein, viscosity

Kruskal-Wallis test	
P value	<0.0001
Exact or approximate P value?	Approximate
P value summary	****
Do the medians vary signif. (P < 0.05)?	Yes
Number of groups	4
Kruskal-Wallis statistic	38.15
Data summary	
Number of treatments (columns)	4
Number of values (total)	72

Multiple Comparisons

Number of families	1					
Number of comparisons per family	3					
Alpha	0.05					
Dunn's multiple comparisons test	Mean rank diff.	Significant?	Summary	Adjusted P Value		
TSB no soni. 100% vs. TSB 30 min. 100%	-22.11	Yes	**	0.0046	A-B	
TSB 30 min. 100% vs. TSB 30 min. 50%	-8.528	No	ns	0.6645	B-C	
TSB 30 min. 100% vs. TSB 30 min. 20%	-19.36	Yes	*	0.0165	B-D	
Test details	Mean rank 1	Mean rank 2	Mean rank diff.	n1	n2	Z
TSB no soni. 100% vs. TSB 30 min. 100%	12.94	35.06	-22.11	18	18	3.170
TSB 30 min. 100% vs. TSB 30 min. 50%	35.06	43.58	-8.528	18	18	1.223
TSB 30 min. 100% vs. TSB 30 min. 20%	35.06	54.42	-19.36	18	18	2.776

pSynF

Table Analyzed	Changing protein, viscosity
Kruskal-Wallis test	
P value	<0.0001
Exact or approximate P value?	Approximate
P value summary	****
Do the medians vary signif. (P < 0.05)?	Yes
Number of groups	4
Kruskal-Wallis statistic	49.57
Data summary	
Number of treatments (columns)	4
Number of values (total)	70

Number of families	1					
Number of comparisons per family	3					
Alpha	0.05					
Dunn's multiple comparisons test	Mean rank diff.	Significant?	Summary	Adjusted P Value		
pSynF no soni. 100% vs. pSynF 30 min. 100%	-22.93	Yes	**	0.0031	E-F	
pSynF 30 min. 100% vs. pSynF 30 min. 50%	-7.069	No	ns	0.9359	F-G	
pSynF 30 min. 100% vs. pSynF 30 min. 20%	-24.13	Yes	**	0.0017	F-H	
Test details	Mean rank 1	Mean rank 2	Mean rank diff.	n1	n2	Z
pSynF no soni. 100% vs. pSynF 30 min. 100%	10.44	33.38	-22.93	18	16	3.280
pSynF 30 min. 100% vs. pSynF 30 min. 50%	33.38	40.44	-7.069	16	18	1.011
pSynF 30 min. 100% vs. pSynF 30 min. 20%	33.38	57.50	-24.13	16	18	3.451

Same volume, different protein:

CFU/mL

TSB no soni. 100%	TSB 30 min. 100%	TSB 30 min. 50%	TSB 30 min. 20%	pSynF no soni. 100%	pSynF 30 min. 100%	pSynF 30 min. 50%	pSynF 30 min. 20%
2000000	750000	1.14e+007	5000000	25200	268000	4400000	4100000
2800000	4400000	1.41e+007	1.47e+007	27600	374500	1000000	600000
2150000	3300000	1.11e+007	1.65e+007	15600	324000	2600000	400000
2500000	5200000	1.08e+007	1.5e+007	13200	196000	850000	1350000
2200000	5300000	9000000	1.47e+007	15600	198000	1000000	1350000
2650000	3500000	9250000	9300000	22200	451500	500000	900000
1200000	4800000	7400000	1.38e+007	8400	12500	15500	85000
840000	7800000	1.3e+007	1.16e+007	8800	23700	33000	117500
1600000	1.32e+007	1.025e+007	1.14e+007	8400	320000	31000	168000
1400000	1.575e+007	1.32e+007	1.59e+007	9600	41000	297000	700000
2100000	1.26e+007	1.38e+007	1.05e+007	10400	38000	40000	1050000
1040000	6200000	6300000	1.26e+007	8000	216000	141000	147000
2050000	1.025e+007	1.68e+007	1.17e+007	24500	189000	165000	1000000
2760000	1.77e+007	3.21e+007	2.25e+007	31500	259000	171000	1000000
3480000	2.28e+007	2.61e+007	1.98e+007	22500	47000	45000	1450000
3180000	7400000	1.05e+008	1.95e+007	21000	63000	32000	1050000
3180000	1.32e+007	1.86e+007	1.225e+007	24600	85000	73000	206500
3420000	3400000	1.56e+007	1.15e+007	20400	59000	225000	195000

Normality and Lognormality Tests:

	TSB no soni. 100%	TSB 30 min. 100%	TSB 30 min. 50%	TSB 30 min. 20%	pSynF no soni. 100%	pSynF 30 min. 100%	pSynF 30 min. 50%	pSynF 30 min. 20%
Test for normal distribution								
D'Agostino & Pearson test								
K2	1.241	2.965	41.30	0.8092	3.746	1.846	26.42	27.17
P value	0.5378	0.2271	<0.0001	0.6672	0.1536	0.3973	<0.0001	<0.0001
Passed normality test (alpha=0.05)?	Yes	Yes	No	Yes	Yes	Yes	No	No
P value summary	ns	ns	****	ns	ns	ns	****	****
Anderson-Darling test								
A2*	0.2343	0.6201	3.417	0.3470	0.5912	0.6075	2.788	1.409
P value	0.7587	0.0898	<0.0001	0.4397	0.1067	0.0967	<0.0001	0.0008
Passed normality test (alpha=0.05)?	Yes	Yes	No	Yes	Yes	Yes	No	No
P value summary	ns	ns	****	ns	ns	ns	****	***
Shapiro-Wilk test								
W	0.9591	0.9162	0.4889	0.9669	0.9132	0.9124	0.6031	0.7171
P value	0.5841	0.1108	<0.0001	0.7387	0.0981	0.0950	<0.0001	0.0001
Passed normality test (alpha=0.05)?	Yes	Yes	No	Yes	Yes	Yes	No	No
P value summary	ns	ns	****	ns	ns	ns	****	***
Kolmogorov-Smirnov test								
KS distance	0.1001	0.1747	0.3422	0.1152	0.1614	0.1923	0.2891	0.2153
P value	>0.1000	>0.1000	<0.0001	>0.1000	>0.1000	0.0771	0.0003	0.0270
Passed normality test (alpha=0.05)?	Yes	Yes	No	Yes	Yes	Yes	No	No
P value summary	ns	ns	****	ns	ns	ns	***	*
Number of values	18	18	18	18	18	18	18	18

Kruskal Wallace ANOVA results **<u>TSB</u>**

Table Analyzed	Same volume diff protein

Kruskal-Wallis test	
P value	<0.0001
Exact or approximate P value?	Approximate
P value summary	****
Do the medians vary signif. (P < 0.05)?	Yes
Number of groups	4
Kruskal-Wallis statistic	42.76
Data summary	
Number of treatments (columns)	4
Number of values (total)	72

Number of families	1					
Number of comparisons per family	3					
Alpha	0.05					
Dunn's multiple comparisons test	Mean rank diff.	Significant?	Summary	Adjusted P Value		
TSB no soni. 100% vs. TSB 30 min. 100%	-24.25	Yes	**	0.0015	A-B	
TSB 30 min. 100% vs. TSB 30 min. 50%	-14.58	No	ns	0.1097	B-C	
TSB 30 min. 100% vs. TSB 30 min. 20%	-15.78	No	ns	0.0711	B-D	
Test details	Mean rank 1	Mean rank 2	Mean rank diff.	n1	n2	Z
TSB no soni. 100% vs. TSB 30 min. 100%	10.72	34.97	-24.25	18	18	3.476
TSB 30 min. 100% vs. TSB 30 min. 50%	34.97	49.56	-14.58	18	18	2.091
TSB 30 min. 100% vs. TSB 30 min. 20%	34.97	50.75	-15.78	18	18	2.262

Kruskal Wallace ANOVA results **pSynF**

Table Analyzed	Same volume diff protein
Kruskal-Wallis test	
P value	<0.0001
Exact or approximate P value?	Approximate
P value summary	****
Do the medians vary signif. (P < 0.05)?	Yes
Number of groups	4
Kruskal-Wallis statistic	42.01
Data summary	
Number of treatments (columns)	4
Number of values (total)	72

Number of families	1					
Number of comparisons per family	3					
Alpha	0.05					
Dunn's multiple comparisons test	Mean rank diff.	Significant?	Summary	Adjusted P Value		
pSynF no soni. 100% vs. pSynF 30 min. 100%	-26.69	Yes	***	0.0004	F-G	
pSynF 30 min. 100% vs. pSynF 30 min. 50%	-4.194	No	ns	>0.9999	G-H	
pSynF 30 min. 100% vs. pSynF 30 min. 20%	-17.28	Yes	*	0.0397	G-I	
Test details	Mean rank 1	Mean rank 2	Mean rank diff.	n1	n2	Z
pSynF no soni. 100% vs. pSynF 30 min. 100%	11.11	37.81	-26.69	18	18	3.827
pSynF 30 min. 100% vs. pSynF 30 min. 50%	37.81	42.00	-4.194	18	18	0.6013
pSynF 30 min. 100% vs. pSynF 30 min. 20%	37.81	55.08	-17.28	18	18	2.477

Diff Vol, same viscosity/protein

CFU/mL:

TSB no soni. 100%	TSB 30 min. 100%	TSB 30 min. 50%	TSB 30 min. 20%	pSynF no soni. 100%	pSynF 30 min. 100%	pSynF 30 min. 50%	pSynF 30 min. 20%
1040000	1700000	780000	2900000	29000	117600	42000	37000
760000	1400000	1120000	1950000	14000	40800	42000	27500
770000	1640000	1600000	1400000	15000	50000	39000	18500
890000	1740000	1280000	1350000	16500	24500	40800	28500
760000	1620000	760000	1400000	12000	50400	33000	55000
1160000	1260000	1600000	1500000	19800	190000	37000	63500
670000	2100000	2400000	2000000	47300	240000	84000	156000
940000	3000000	2240000	2650000	36600	960000	73200	364000
1150000	2340000	2520000	3450000	25600	290000	88000	352000
1060000	2900000	2200000	3250000	35400	610000	81000	200000
1430000	3300000	2840000	2700000	34200	110000	93600	75000
1400000	2350000	1720000	1900000	33000	1410000	340000	750000
2100000	1900000	3300000	4200000	25000	49000	88200	78000
1680000	2520000	2500000	3000000	28800	124800	48000	78000
1840000	2150000	3100000	4000000	17600	82500	67200	46000
1640000	2650000	3400000	5750000	25000	107800	43200	122000
1740000	2900000	3100000	2400000	27500	62100	174600	515000
1320000	2300000	3200000	3400000	21000	140000	40000	198000

Normality/Lognormality

	TSB no soni. 100%	TSB 30 min. 100%	TSB 30 min. 50%	TSB 30 min. 20%	pSynF no soni. 100%	pSynF 30 min. 100%	pSynF 30 min. 50%	pSynF 30 min. 20%
Test for normal distribution								
D'Agostino & Pearson test								
K2	1.529	1.146	2.672	4.432	1.035	21.52	31.60	13.93
P value	0.4656	0.5639	0.2630	0.1090	0.5961	<0.0001	<0.0001	0.0009
Passed normality test (alpha=0.05)?	Yes	Yes	Yes	Yes	Yes	No	No	No
P value summary	ns	ns	ns	ns	ns	****	****	***
Anderson-Darling test								
A2*	0.3474	0.2196	0.3752	0.3735	0.2168	2.730	2.449	1.637
P value	0.4385	0.8061	0.3754	0.3791	0.8147	<0.0001	<0.0001	0.0002
Passed normality test (alpha=0.05)?	Yes	Yes	Yes	Yes	Yes	No	No	No
P value summary	ns	ns	ns	ns	ns	****	****	***
Shapiro-Wilk test								
W	0.9451	0.9688	0.9342	0.9235	0.9642	0.6332	0.6110	0.7600
P value	0.3534	0.7746	0.2298	0.1489	0.6837	<0.0001	<0.0001	0.0004
Passed normality test (alpha=0.05)?	Yes	Yes	Yes	Yes	Yes	No	No	No
P value summary	ns	ns	ns	ns	ns	****	****	***
Kolmogorov-Smirnov test								
KS distance	0.1317	0.1201	0.1255	0.1241	0.08658	0.2999	0.3197	0.2435
P value	>0.1000	>0.1000	>0.1000	>0.1000	>0.1000	0.0001	<0.0001	0.0060
Passed normality test (alpha=0.05)?	Yes	Yes	Yes	Yes	Yes	No	No	No
P value summary	ns	ns	ns	ns	ns	***	****	**
Number of values	18	18	18	18	18	18	18	18

Kruskal Wallis test

TSB

Table Analyzed	diff vol same viscosity
Kruskal-Wallis test	
P value	<0.0001

Exact or approximate P value?	Approximate
P value summary	****
Do the medians vary signif. (P < 0.05)?	Yes
Number of groups	4
Kruskal-Wallis statistic	24.39
Data summary	
Number of treatments (columns)	4
Number of values (total)	72

Number of families	1					
Number of comparisons per family	3					
Alpha	0.05					
Dunn's multiple comparisons test	Mean rank diff.	Significant?	Summary	Adjusted P Value		
TSB no soni. 100% vs. TSB 30 min. 100%	-25.08	Yes	***	0.0010	A-B	
TSB 30 min. 100% vs. TSB 30 min. 50%	1.194	No	ns	>0.9999	B-C	
TSB 30 min. 100% vs. TSB 30 min. 20%	-7.278	No	ns	0.8902	B-D	
Test details	Mean rank 1	Mean rank 2	Mean rank diff.	n1	n2	Z
TSB no soni. 100% vs. TSB 30 min. 100%	16.17	41.25	-25.08	18	18	3.596
TSB 30 min. 100% vs. TSB 30 min. 50%	41.25	40.06	1.194	18	18	0.1713
TSB 30 min. 100% vs. TSB 30 min. 20%	41.25	48.53	-7.278	18	18	1.043

pSynF

Table Analyzed	diff vol same viscosity
Kruskal-Wallis test	
P value	<0.0001
Exact or approximate P value?	Approximate
P value summary	****
Do the medians vary signif. (P < 0.05)?	Yes
Number of groups	4
Kruskal-Wallis statistic	34.41
Data summary	
Number of treatments (columns)	4
Number of values (total)	72

Number of families	1					
Number of comparisons per family	3					
Alpha	0.05					
Dunn's multiple comparisons test	Mean rank diff.	Significant?	Summary	Adjusted P Value		
pSynF no soni. 100% vs. pSynF 30 min. 100%	-37.64	Yes	****	<0.0001	F-G	
pSynF 30 min. 100% vs. pSynF 30 min. 50%	11.44	No	ns	0.3026	G-H	
pSynF 30 min. 100% vs. pSynF 30 min. 20%	5.250	No	ns	>0.9999	G-I	
Test details	Mean rank 1	Mean rank 2	Mean rank diff.	n1	n2	Z
pSynF no soni. 100% vs. pSynF 30 min. 100%	12.44	50.08	-37.64	18	18	5.396
pSynF 30 min. 100% vs. pSynF 30 min. 50%	50.08	38.64	11.44	18	18	1.641
pSynF 30 min. 100% vs. pSynF 30 min. 20%	50.08	44.83	5.250	18	18	0.7526

Fig. 3B

20% TSB

Alamar Blue fluorescence

	no sonication		30 min sonication	
	0 min	2h	0min	2h
	900	9968	828	11249
	817	10359	858	9108
	818	9459	833	11242
	808	9853	735	9845
	809	9377	855	10175
	824	8203	842	8912
	750	8233	851	8408
	849	7947	843	8741
	741	8405	811	10238
	839	8168	789	10480
	751	8111	779	8298
	827	8510	833	9393
mean	811.0833	8882.75	821.4167	9674.083
ratio		10.95171		11.77732

Mann-Whitney test for non-parametric data

	0 min sonication preincubation presonication	0 min sonication 2h incubation 37 °C	0 min sonication preincubation presonication	after 30 min sonication 2h incubation 37 °C
Test for normal distribution				
D'Agostino & Pearson test				
K2	0.1463	3.060	5.811	1.311
P value	0.9294	0.2165	0.0547	0.5193
Passed normality test (alpha=0.05)?	Yes	Yes	Yes	Yes
P value summary	ns	ns	ns	ns
Anderson-Darling test				
A2*	0.5208	0.7579	0.6638	0.2616
P value	0.1466	0.0348	0.0619	0.6379
Passed normality test (alpha=0.05)?	Yes	No	Yes	Yes
P value summary	ns	*	ns	ns
Shapiro-Wilk test				
W	0.9189	0.8606	0.8610	0.9378
P value	0.2772	0.0498	0.0504	0.4702
Passed normality test (alpha=0.05)?	Yes	No	Yes	Yes
P value summary	ns	*	ns	ns
Kolmogorov-Smirnov test				
KS distance	0.2231	0.2512	0.2375	0.1265

P value	>0.1000	0.0351	0.0601	>0.1000
Passed normality test (alpha=0.05)?	Yes	No	Yes	Yes
P value summary	ns	*	ns	ns
Number of values	12	12	12	12

Table Analyzed	For statistics
Kruskal-Wallis test	
P value	<0.0001
Exact or approximate P value?	Approximate
P value summary	****
Do the medians vary signif. (P < 0.05)?	Yes
Number of groups	4
Kruskal-Wallis statistic	36.62
Data summary	
Number of treatments (columns)	4
Number of values (total)	48

Number of families	1					
Number of comparisons per family	4					
Alpha	0.05					
Dunn's multiple comparisons test	Mean rank diff.	Significant?	Summary	Adjusted P Value		
Column A vs. Column B	-22.67	Yes	***	0.0003	A-B	
Column A vs. Column D	-3.167	No	ns	>0.9999	A-D	
Column B vs. Column E	-5.833	No	ns	>0.9999	B-E	
Column D vs. Column E	-25.33	Yes	****	<0.0001	D-E	
Test details	Mean rank 1	Mean rank 2	Mean rank diff.	n1	n2	Z
Column A vs. Column B	10.92	33.58	-22.67	12	12	3.966
Column A vs. Column D	10.92	14.08	-3.167	12	12	0.5541
Column B vs. Column E	33.58	39.42	-5.833	12	12	1.021
Column D vs. Column E	14.08	39.42	-25.33	12	12	4.433

20% pSynF

	no sonication		30 min sonication	
	0 min	2h	0min	2h
	407	2238	566	9456
	604	5596	597	6489
	597	4670	624	13784
	475	3126	543	13989
	452	2986	600	15764
	599	2748	581	9244
	164	1032	527	8764
	572	4745	561	5902
	474	3400	541	19604
	562	3181	593	22366
	608	4806	579	40324
	637	4792	555	10532
mean	512.5833	3610	572.25	14684.83
ratio		7.042757		25.66157

	0 min sonication preincubation presonication	0 min sonication 2h incubation 37 °C	30 min sonication preincubation presonication	30 min sonication 2h incubation 37 °C
Test for normal distribution				
D'Agostino & Pearson test				
K2	12.30	0.3126	0.2204	14.37
P value	0.0021	0.8553	0.8956	0.0008
Passed normality test (alpha=0.05)?	No	Yes	Yes	No
P value summary	**	ns	ns	***
Anderson-Darling test				
A2*	0.8783	0.4034	0.1520	0.8521
P value	0.0167	0.2999	0.9433	0.0195
Passed normality test (alpha=0.05)?	No	Yes	Yes	No
P value summary	*	ns	ns	*
Shapiro-Wilk test				
W	0.8008	0.9403	0.9796	0.7991
P value	0.0096	0.5016	0.9821	0.0092
Passed normality test (alpha=0.05)?	No	Yes	Yes	No
P value summary	**	ns	ns	**
Kolmogorov-Smirnov test				
KS distance	0.2289	0.2048	0.09978	0.2049
P value	0.0826	>0.1000	>0.1000	>0.1000
Passed normality test (alpha=0.05)?	Yes	Yes	Yes	Yes
P value summary	ns	ns	ns	ns
Number of values	12	12	12	12

Table Analyzed	For statistics
Kruskal-Wallis test	
P value	<0.0001
Exact or approximate P value?	Approximate
P value summary	****
Do the medians vary signif. (P < 0.05)?	Yes
Number of groups	4
Kruskal-Wallis statistic	39.72
Data summary	
Number of treatments (columns)	4
Number of values (total)	48

Number of families	1					
Number of comparisons per family	4					
Alpha	0.05					
Dunn's multiple comparisons test	Mean rank diff.	Significant?	Summary	Adjusted P Value		
Column A vs. Column B	-18.63	Yes	**	0.0045	A-B	
Column A vs. Column D	-1.250	No	ns	>0.9999	A-D	
Column B vs. Column E	-12.00	No	ns	0.1430	B-E	
Column D vs. Column E	-29.38	Yes	****	<0.0001	D-E	
Test details	Mean rank 1	Mean rank 2	Mean rank diff.	n1	n2	Z
Column A vs. Column B	11.88	30.50	-18.63	12	12	3.259
Column A vs. Column D	11.88	13.13	-1.250	12	12	0.2187
Column B vs. Column E	30.50	42.50	-12.00	12	12	2.100
Column D vs. Column E	13.13	42.50	-29.38	12	12	5.140

Fig. 3C

TSB

CFU/mL (trypsin)

0 µg/mL AMK	30 ug/mL AMK	0 µg/mL AMK+US	30 ug/mL AMK+US
8.8e+008	1560	1.3e+009	1140
5.4e+008	2300	1.14e+009	1340
6.6e+008	1650	1.46e+009	1720
8.4e+008	1840	1.26e+009	1600
8.2e+008	1560	1.28e+009	150
6.8e+008	1850	1.58e+009	200
8.8e+008	2150	1e+009	1
1.12e+009	1840	1.68e+009	1
1.16e+009	520	1.1e+009	70
1.2e+009	270	1.38e+009	110
1e+009	2200	1.34e+009	180
1.28e+009	1360	1.48e+009	500

Normality and Lognormality

	0 µg/mL AMK	30 ug/mL AMK	0 µg/mL AMK+US	30 ug/mL AMK+US
Test for normal distribution				
D'Agostino & Pearson test				
K2	0.8347	4.401	0.04088	2.982
P value	0.6588	0.1107	0.9798	0.2252
Passed normality test (alpha=0.05)?	Yes	Yes	Yes	Yes
P value summary	ns	ns	ns	ns
Anderson-Darling test				
A2*	0.2348	0.6914	0.1206	1.100
P value	0.7344	0.0523	0.9829	0.0043
Passed normality test (alpha=0.05)?	Yes	Yes	Yes	No
P value summary	ns	ns	ns	**
Shapiro-Wilk test				
W	0.9601	0.8634	0.9872	0.7934
P value	0.7850	0.0539	0.9987	0.0079
Passed normality test (alpha=0.05)?	Yes	Yes	Yes	No
P value summary	ns	ns	ns	**
Kolmogorov-Smirnov test				
KS distance	0.1541	0.2299	0.1058	0.3014
P value	>0.1000	0.0797	>0.1000	0.0035
Passed normality test (alpha=0.05)?	Yes	Yes	Yes	No
P value summary	ns	ns	ns	**
Number of values	12	12	12	12

Mann-Whitney for comparison of two means with non-parametric data.

Table Analyzed	TSB
Column B	30 ug/mL AMK
vs.	vs.
Column A	0 µg/mL AMK
Mann Whitney test	
P value	<0.0001
Exact or approximate P value?	Exact
P value summary	****
Significantly different (P < 0.05)?	Yes

One- or two-tailed P value?	Two-tailed
Sum of ranks in column A,B	222 , 78
Mann-Whitney U	0
Difference between medians	
Median of column A	880000000, n=12
Median of column B	1745, n=12
Difference: Actual	-879998255
Difference: Hodges-Lehmann	-879998255

Table Analyzed	TSB
Column D	30 ug/mL AMK+US
vs.	vs.
Column C	0 µg/mL AMK+US
Mann Whitney test	
P value	<0.0001
Exact or approximate P value?	Exact
P value summary	****
Significantly different (P < 0.05)?	Yes
One- or two-tailed P value?	Two-tailed
Sum of ranks in column C,D	222 , 78
Mann-Whitney U	0
Difference between medians	
Median of column C	1320000000, n=12
Median of column D	190.0, n=12
Difference: Actual	-1319999810
Difference: Hodges-Lehmann	-1319999140

Table Analyzed	TSB
Column D	30 ug/mL AMK+US
vs.	vs.
Column B	30 ug/mL AMK
Mann Whitney test	
P value	0.0006
Exact or approximate P value?	Exact
P value summary	***
Significantly different (P < 0.05)?	Yes
One- or two-tailed P value?	Two-tailed
Sum of ranks in column B,D	206 , 94
Mann-Whitney U	16
Difference between medians	
Median of column B	1745, n=12
Median of column D	190.0, n=12
Difference: Actual	-1555
Difference: Hodges-Lehmann	-1270

pSynF

CFU/mL (trypsin)

0 µg/mL AMK	30 ug/mL AMK	0 µg/mL AMK+US	30 ug/mL AMK+US	20% 0 µg/mL AMK	20%30 ug/mL AMK	20%0 µg/mL AMK+US	20%30 ug/mL AMK+US
2e+008	2.3e+007	4.3e+008	2.68e+007	1.6e+008	1.1e+007	6.9e+008	2.3e+007
2.3e+008	2.55e+007	5.1e+008	2.52e+007	5.4e+007	1.52e+007	9.9e+008	2.65e+007
2.1e+008	2.2e+007	3e+008	3.18e+007	3.4e+007	1.52e+007	5.5e+008	2.75e+007
1.6e+008	2.9e+007	4.8e+008	5.5e+007	3.78e+007	1.14e+007	6.1e+008	4.6e+007
1.8e+008	3e+007	3.4e+008	4.2e+007	2.04e+007	2.34e+007	4.4e+008	1.98e+007
2.6e+008	3.2e+007	8.6e+008	4.44e+007	2.25e+007	1.6e+007	3.9e+008	8200000
3.8e+008	2.35e+007	5.4e+008	4.44e+007	2.4e+007	1.28e+007	6.7e+008	4.2e+007
1.7e+008	3.66e+007	8.2e+008	3.1e+007	3e+007	1.28e+007	3.6e+007	1.28e+007
2.7e+008	7800000	5.9e+008	2.4e+007	4.3e+007	1.34e+007	2.7e+008	2.4e+007
2.2e+008	1.48e+007	1.08e+009	2e+007	2.97e+007	1e+007	3.5e+008	3200000
3.48e+007	6000000	4.1e+008	3.05e+007	3e+007	1.9e+007	8.2e+008	2600000
3.2e+007	8400000	4.9e+008	2.3e+007	3.12e+007	1.65e+007	3.1e+008	2.64e+007

Normality and lognormality test

	0 µg/mL AMK	30 ug/mL AMK	0 µg/mL AMK+US	30 ug/mL AMK+US	20% 0 µg/mL AMK	20%30 ug/mL AMK	20%0 µg/mL AMK+US	20%30 ug/mL AMK+US
Test for normal distribution								
D'Agostino & Pearson test								
K2	0.8551	1.312	3.653	1.573	29.35	4.338	0.04118	0.1955
P value	0.6521	0.5190	0.1610	0.4554	<0.0001	0.1143	0.9796	0.9069
Passed normality test (alpha=0.05)?	Yes	Yes	Yes	Yes	No	Yes	Yes	Yes
P value summary	ns	ns	ns	ns	****	ns	ns	ns
Anderson-Darling test								
A2*	0.4408	0.3744	0.6156	0.4932	2.236	0.3569	0.1378	0.3192
P value	0.2399	0.3555	0.0831	0.1742	<0.0001	0.3932	0.9648	0.4876
Passed normality test (alpha=0.05)?	Yes	Yes	Yes	Yes	No	Yes	Yes	Yes
P value summary	ns	ns	ns	ns	****	ns	ns	ns
Shapiro-Wilk test								
W	0.9302	0.9294	0.8868	0.9103	0.5457	0.9244	0.9889	0.9399
P value	0.3818	0.3742	0.1072	0.2151	<0.0001	0.3247	0.9995	0.4974
Passed normality test (alpha=0.05)?	Yes	Yes	Yes	Yes	No	Yes	Yes	Yes
P value summary	ns	ns	ns	ns	****	ns	ns	ns
Kolmogorov-Smirnov test								
KS distance	0.1882	0.1843	0.2193	0.2173	0.3339	0.1516	0.1055	0.1727
P value	>0.1000	>0.1000	>0.1000	>0.1000	0.0006	>0.1000	>0.1000	>0.1000
Passed normality test (alpha=0.05)?	Yes	Yes	Yes	Yes	No	Yes	Yes	Yes
P value summary	ns	ns	ns	ns	***	ns	ns	ns
Number of values	12	12	12	12	12	12	12	12

Mann-Whitney Tests

Table Analyzed	pSynF
Column B	30 ug/mL AMK

vs.	vs.
Column A	0 µg/mL AMK
Mann Whitney test	
P value	<0.0001
Exact or approximate P value?	Exact
P value summary	****
Significantly different (P < 0.05)?	Yes
One- or two-tailed P value?	Two-tailed
Sum of ranks in column A,B	219.5 , 80.50
Mann-Whitney U	2.500
Difference between medians	
Median of column A	205000000, n=12
Median of column B	23250000, n=12
Difference: Actual	-181750000
Difference: Hodges-Lehmann	-182200000

Table Analyzed	pSynF
Column D	30 ug/mL AMK+US
vs.	vs.
Column C	0 µg/mL AMK+US
Mann Whitney test	
P value	<0.0001
Exact or approximate P value?	Exact
P value summary	****
Significantly different (P < 0.05)?	Yes
One- or two-tailed P value?	Two-tailed
Sum of ranks in column C,D	222 , 78
Mann-Whitney U	0
Difference between medians	
Median of column C	500000000, n=12
Median of column D	30750000, n=12
Difference: Actual	-469250000
Difference: Hodges-Lehmann	-465800000

Table Analyzed	pSynF
Column F	20%30 ug/mL AMK
vs.	vs.
Column E	20% 0 µg/mL AMK
Mann Whitney test	
P value	<0.0001
Exact or approximate P value?	Exact
P value summary	****
Significantly different (P < 0.05)?	Yes
One- or two-tailed P value?	Two-tailed
Sum of ranks in column E,F	220 , 80
Mann-Whitney U	2
Difference between medians	
Median of column E	30600000, n=12
Median of column F	14300000, n=12
Difference: Actual	-16300000
Difference: Hodges-Lehmann	-17350000

Table Analyzed	pSynF
Column H	20%30 ug/mL AMK+US
vs.	vs.

Column G	20%0 μg/mL AMK+US
Mann Whitney test	
P value	<0.0001
Exact or approximate P value?	Exact
P value summary	****
Significantly different (P < 0.05)?	Yes
One- or two-tailed P value?	Two-tailed
Sum of ranks in column G,H	220 , 80
Mann-Whitney U	2
Difference between medians	
Median of column G	495000000, n=12
Median of column H	23500000, n=12
Difference: Actual	-471500000
Difference: Hodges-Lehmann	-470700000

Table Analyzed	pSynF
Column D	30 ug/mL AMK+US
vs.	vs.
Column B	30 ug/mL AMK
Mann Whitney test	
P value	0.0295
Exact or approximate P value?	Exact
P value summary	*
Significantly different (P < 0.05)?	Yes
One- or two-tailed P value?	Two-tailed
Sum of ranks in column B,D	112.5 , 187.5
Mann-Whitney U	34.50
Difference between medians	
Median of column B	23250000, n=12
Median of column D	30750000, n=12
Difference: Actual	7500000
Difference: Hodges-Lehmann	11000000

Table Analyzed	pSynF
Column H	20%30 ug/mL AMK+US
vs.	vs.
Column F	20%30 ug/mL AMK
Mann Whitney test	
P value	0.1386
Exact or approximate P value?	Exact
P value summary	ns
Significantly different (P < 0.05)?	No
One- or two-tailed P value?	Two-tailed
Sum of ranks in column F,H	124 , 176
Mann-Whitney U	46
Difference between medians	
Median of column F	14300000, n=12
Median of column H	23500000, n=12
Difference: Actual	9200000
Difference: Hodges-Lehmann	8200000

SynF

CFU/mL (trypsin)

0 μg/mL AMK	30 ug/mL AMK	0 μg/mL AMK+US	30 ug/mL AMK+US	20% 0 μg/mL AMK	20%30 ug/mL AMK	20%0 μg/mL AMK+US	20%30 ug/mL AMK+US
3.7e+007	680	4.9e+007	1	3.4e+007	2500	1.18e+008	1
2.5e+007	4800	3.7e+007	1	3.5e+007	4200	1.16e+008	1
3e+007	42700	4.1e+007	1	3e+007	1200	9.4e+007	1
3.3e+007	180000	3.7e+007	1	2.1e+007	17600	8e+007	1
3.06e+008	5.5e+007	1.9e+007	1	7.8e+007	2000	3.4e+007	1
1.55e+008	5.5e+007	1.3e+007	1	4.7e+007	29000	3e+007	1.1
2.7e+008	5.4e+007	1.8e+007	1	4.8e+007	21500	2.9e+007	1.01
2e+008	5.7e+007	2e+007	1	6.3e+007	700	3.2e+007	1
5.6e+007	160000	3.8e+007	520	5.9e+007	7000	1.16e+008	1
4.8e+007	2350000	2.5e+007	260	9.8e+007	8200	5.6e+007	1
5.8e+007	37800	2.7e+007	140	1e+008	5100	4e+007	1
4.4e+007	23000	2.4e+007	1	5.7e+007	8300	1.34e+008	1

Normality and lognormality

	0 μg/mL AMK	30 ug/mL AMK	0 μg/mL AMK+US	30 ug/mL AMK+US	20% 0 μg/mL AMK	20%30 ug/mL AMK	20%0 μg/mL AMK+US	20%30 ug/mL AMK+US
Test for normal distribution								
D'Agostino & Pearson test								
K2	3.457	5.292	1.020	17.67	1.036	4.922	5.540	32.93
P value	0.1775	0.0709	0.6006	0.0001	0.5958	0.0854	0.0627	<0.0001
Passed normality test (alpha=0.05)?	Yes	Yes	Yes	No	Yes	Yes	Yes	No
P value summary	ns	ns	ns	***	ns	ns	ns	****
Anderson-Darling test								
A2*	1.289	2.180	0.3571	2.402	0.3266	0.8540	0.6993	3.606
P value	0.0014	<0.0001	0.3929	<0.0001	0.4676	0.0193	0.0498	<0.0001
Passed normality test (alpha=0.05)?	No	No	Yes	No	Yes	No	No	No
P value summary	**	****	ns	****	ns	*	*	****
Shapiro-Wilk test								
W	0.7632	0.6295	0.9439	0.5681	0.9324	0.8302	0.8566	0.3709
P value	0.0037	0.0002	0.5500	<0.0001	0.4063	0.0211	0.0443	<0.0001
Passed normality test (alpha=0.05)?	No	No	Yes	No	Yes	No	No	No
P value summary	**	***	ns	****	ns	*	*	****
Kolmogorov-Smirnov test								
KS distance	0.3462	0.3933	0.1811	0.4323	0.1398	0.2784	0.2092	0.4584
P value	0.0003	<0.0001	>0.1000	<0.0001	>0.1000	0.0108	>0.1000	<0.0001
Passed normality test (alpha=0.05)?	No	No	Yes	No	Yes	No	Yes	No
P value summary	***	****	ns	****	ns	*	ns	****
Number of values	12	12	12	12	12	12	12	12

Use Mann-Whitney tests as anova over correcting for column with only 1’s

Table Analyzed	SynF
Column B	30 ug/mL AMK

vs.	vs.
Column A	0 μg/mL AMK
Mann Whitney test	
P value	0.0053
Exact or approximate P value?	Exact
P value summary	**
Significantly different (P < 0.05)?	Yes
One- or two-tailed P value?	Two-tailed
Sum of ranks in column A,B	197 , 103
Mann-Whitney U	25
Difference between medians	
Median of column A	52000000, n=12
Median of column B	170000, n=12
Difference: Actual	-51830000
Difference: Hodges-Lehmann	-44824660

Table Analyzed	SynF
Column D	30 ug/mL AMK+US
vs.	vs.
Column C	0 μg/mL AMK+US
Mann Whitney test	
P value	<0.0001
Exact or approximate P value?	Exact
P value summary	****
Significantly different (P < 0.05)?	Yes
One- or two-tailed P value?	Two-tailed
Sum of ranks in column C,D	222 , 78
Mann-Whitney U	0
Difference between medians	
Median of column C	26000000, n=12
Median of column D	1.000, n=12
Difference: Actual	-25999999
Difference: Hodges-Lehmann	-25999740

Table Analyzed	SynF
Column F	20%30 ug/mL AMK
vs.	vs.
Column E	20% 0 μg/mL AMK
Mann Whitney test	
P value	<0.0001
Exact or approximate P value?	Exact
P value summary	****
Significantly different (P < 0.05)?	Yes
One- or two-tailed P value?	Two-tailed
Sum of ranks in column E,F	222 , 78
Mann-Whitney U	0
Difference between medians	
Median of column E	52500000, n=12
Median of column F	6050, n=12
Difference: Actual	-52493950
Difference: Hodges-Lehmann	-52485150

Table Analyzed	SynF
Column H	20%30 ug/mL AMK+US
vs.	vs.

Column G	20%0 µg/mL AMK+US
Mann Whitney test	
P value	<0.0001
Exact or approximate P value?	Exact
P value summary	****
Significantly different (P < 0.05)?	Yes
One- or two-tailed P value?	Two-tailed
Sum of ranks in column G,H	222 , 78
Mann-Whitney U	0
Difference between medians	
Median of column G	68000000, n=12
Median of column H	1.000, n=12
Difference: Actual	-67999999
Difference: Hodges-Lehmann	-67999999

Table Analyzed	SynF
Column D	30 ug/mL AMK+US
vs.	vs.
Column B	30 ug/mL AMK
Mann Whitney test	
P value	<0.0001
Exact or approximate P value?	Exact
P value summary	****
Significantly different (P < 0.05)?	Yes
One- or two-tailed P value?	Two-tailed
Sum of ranks in column B,D	222 , 78
Mann-Whitney U	0
Difference between medians	
Median of column B	170000, n=12
Median of column D	1.000, n=12
Difference: Actual	-169999
Difference: Hodges-Lehmann	-169740

Table Analyzed	SynF
Column H	20%30 ug/mL AMK+US
vs.	vs.
Column F	20%30 ug/mL AMK
Mann Whitney test	
P value	<0.0001
Exact or approximate P value?	Exact
P value summary	****
Significantly different (P < 0.05)?	Yes
One- or two-tailed P value?	Two-tailed
Sum of ranks in column F,H	222 , 78
Mann-Whitney U	0
Difference between medians	
Median of column F	6050, n=12
Median of column H	1.000, n=12
Difference: Actual	-6049
Difference: Hodges-Lehmann	-6049

Fig. 4A

Alamar blue for the different combinations, measured in fluorescence

20% TSB

preincubation preultrasound	2h incubation 37 °C	
1046	12965	
1085	11759	
1066	12093	
1089	11755	
1001	10407	
1015	10854	
1018	10367	
1053	11768	
792	10282	
839	10023	
881	10150	
938	8652	
726	8131	
776	8808	
743	8495	
740	7569	
925.5	10254.875	Average
137.1772576	1580.517758	S. D.
	11.08036197	Ratio

20% TSB US mcbl

preincubation preultrasound	2h incubation 37 °C	
1087	14168	
1042	13624	
1044	12124	
1106	12065	
1105	12808	
1088	13249	
1032	12859	
1042	11852	
897	9876	
889	9878	
926	9191	
922	9147	
730	7777	
720	7388	
735	7007	
786	7346	
946.9375	10867.53	Average
141.6152	2473.829	S. D.
	11.4765054	Ratio

Normality, lognormality for all TSB values

	preincubation preultrasound	2h incubation 37 °C		US preincubation preultrasound	US 2h incubation 37 °C
Test for normal distribution					
D'Agostino & Pearson test					
K2	7.302	0.9449		3.262	4.820
P value	0.0260	0.6235		0.1958	0.0898
Passed normality test (alpha=0.05)?	No	Yes		Yes	Yes
P value summary	*	ns		ns	ns
Anderson-Darling test					
A2*	0.7685	0.3313		0.7527	0.5456
P value	0.0360	0.4734		0.0395	0.1347
Passed normality test (alpha=0.05)?	No	Yes		No	Yes
P value summary	*	ns		*	ns
Shapiro-Wilk test					
W	0.8720	0.9563		0.8717	0.9105
P value	0.0292	0.5963		0.0289	0.1185
Passed normality test (alpha=0.05)?	No	Yes		No	Yes
P value summary	*	ns		*	ns
Kolmogorov-Smirnov test					
KS distance	0.2090	0.1412		0.2260	0.1868
P value	0.0601	>0.1000		0.0283	>0.1000
Passed normality test (alpha=0.05)?	Yes	Yes		No	Yes
P value summary	ns	ns		*	ns
Number of values	16	16		16	16

ANOVA with Kruskal-Wallis test as non-parametric

Table Analyzed	20% TSB all
Kruskal-Wallis test	
P value	<0.0001
Exact or approximate P value?	Approximate
P value summary	****
Do the medians vary signif. (P < 0.05)?	Yes
Number of groups	4
Kruskal-Wallis statistic	47.39
Data summary	
Number of treatments (columns)	4
Number of values (total)	64

Number of families	1					
Number of comparisons per family	3					
Alpha	0.05					
Dunn's multiple comparisons test	Mean rank diff.	Significant?	Summary	Adjusted P Value		
preincubation preultrasound vs. 2h incubation 37 °C	-31.88	Yes	****	<0.0001	A-B	
2h incubation 37 °C vs. US 2h incubation 37 °C	-1.750	No	ns	>0.9999	B-E	
US preincubation preultrasound vs. US 2h incubation 37 °C	-32.13	Yes	****	<0.0001	D-E	
Test details	Mean rank 1	Mean rank 2	Mean rank diff.	n1	n2	Z
preincubation preultrasound vs. 2h incubation 37 °C	15.75	47.63	-31.88	16	16	4.842
2h incubation 37 °C vs. US 2h incubation 37 °C	47.63	49.38	-1.750	16	16	0.2658
US preincubation preultrasound vs. US 2h incubation 37 °C	17.25	49.38	-32.13	16	16	4.880

20% pSynF

preincubation preultrasound	2h incubation 37 °C	
657	3651	
603	1446	
568	2129	
587	2097	
637	1743	
623	2761	
600	2236	
575	1714	
543	4419	
595	5018	
530	6276	
557	5496	
547	7816	
589	5290	
648	6559	
650	5424	
594.3125	4004.688	Average
40.17747	2035.77	S. D.
	6.738353	Ratio

20% pSynF US mcbl

preincubation preultrasound	2h incubation 37 °C	
614	3628	
634	3870	
541	3542	
561	6232	
568	4109	
605	3897	
580	4666	
609	4405	
707	5395	
631	6105	
616	6185	
646	6449	
623	7909	
696	9123	
602	7383	
625	8556	
616.125	5715.875	Average
43.87843	1821.088	S. D.
	9.277135	Ratio

Normality and lognormality, pSynF

	preincubation preultrasound	2h incubation 37 °C		US preincubation preultrasound	US 2h incubation 37 °C

Test for normal distribution					
D'Agostino & Pearson test					
K2	1.463	2.520		1.319	1.760
P value	0.4812	0.2837		0.5172	0.4148
Passed normality test (alpha=0.05)?	Yes	Yes		Yes	Yes
P value summary	ns	ns		ns	ns
Anderson-Darling test					
A2*	0.2414	0.5391		0.3858	0.4515
P value	0.7289	0.1403		0.3486	0.2380
Passed normality test (alpha=0.05)?	Yes	Yes		Yes	Yes
P value summary	ns	ns		ns	ns
Shapiro-Wilk test					
W	0.9547	0.9168		0.9492	0.9196
P value	0.5678	0.1500		0.4777	0.1665
Passed normality test (alpha=0.05)?	Yes	Yes		Yes	Yes
P value summary	ns	ns		ns	ns
Kolmogorov-Smirnov test					
KS distance	0.1060	0.1825		0.1544	0.1554
P value	>0.1000	>0.1000		>0.1000	>0.1000
Passed normality test (alpha=0.05)?	Yes	Yes		Yes	Yes
P value summary	ns	ns		ns	ns
Number of values	16	16		16	16

ANOVA with Kruskal-Wallis test as non-parametric

Table Analyzed	20% pSynF all
Kruskal-Wallis test	
P value	<0.0001
Exact or approximate P value?	Approximate
P value summary	****
Do the medians vary signif. (P < 0.05)?	Yes
Number of groups	4
Kruskal-Wallis statistic	48.73
Data summary	
Number of treatments (columns)	4
Number of values (total)	64

Number of families	1					
Number of comparisons per family	3					
Alpha	0.05					
Dunn's multiple comparisons test	Mean rank diff.	Significant?	Summary	Adjusted P Value		
preincubation preultrasound vs. 2h incubation 37 °C	-30.75	Yes	****	<0.0001	A-B	
2h incubation 37 °C vs. US 2h incubation 37 °C	-6.750	No	ns	0.9155	B-E	
US preincubation preultrasound vs. US 2h incubation 37 °C	-33.25	Yes	****	<0.0001	D-E	
Test details	Mean rank 1	Mean rank 2	Mean rank diff.	n1	n2	Z
preincubation preultrasound vs. 2h incubation 37 °C	14.38	45.13	-30.75	16	16	4.671
2h incubation 37 °C vs. US 2h incubation 37 °C	45.13	51.88	-6.750	16	16	1.025
US preincubation preultrasound vs. US 2h incubation 37 °C	18.63	51.88	-33.25	16	16	5.051

20% SynF

preincubation preultrasound	2h incubation 37 °C	
487	1269	
466	603	
482	517	
476	707	
447	572	
495	526	
541	521	
459	556	
555	506	
508	517	
433	561	
644	548	
499.4167	616.9167	Average
57.78559	212.5304	S. D.
	1.235274	Ratio

20% SynF US mcbl

preincubation preultrasound	2h incubation 37 °C	
371	1698	
390	538	
453	1463	
355	579	
340	583	
352	589	
357	576	
323	543	
349	618	
320	569	
343	675	
393	565	
362.1667	749.6667	Average
36.40638	392.9176	S. D.
	2.069949	Ratio

Normality and lognormality

	preincubation preultrasound	2h incubation 37 °C		US preincubation preultrasound	US 2h incubation 37 °C
Test for normal distribution					
D'Agostino & Pearson test					
K2	8.778	28.96		8.305	12.92
P value	0.0124	<0.0001		0.0157	0.0016
Passed normality test (alpha=0.05)?	No	No		No	No
P value summary	*	****		*	**
Anderson-Darling test					
A2*	0.5742	2.360		0.5630	2.576
P value	0.1072	<0.0001		0.1148	<0.0001
Passed normality test (alpha=0.05)?	Yes	No		Yes	No
P value summary	ns	****		ns	****

Shapiro-Wilk test					
W	0.8746	0.5289		0.8773	0.5608
P value	0.0749	<0.0001		0.0809	<0.0001
Passed normality test (alpha=0.05)?	Yes	No		Yes	No
P value summary	ns	****		ns	****
Kolmogorov-Smirnov test					
KS distance	0.1971	0.3594		0.2231	0.4087
P value	>0.1000	0.0001		>0.1000	<0.0001
Passed normality test (alpha=0.05)?	Yes	No		Yes	No
P value summary	ns	***		ns	****
Number of values	12	12		12	12

ANOVA with Kruskal-Wallis test as non-parametric

Table Analyzed	20% SynF all
Kruskal-Wallis test	
P value	<0.0001
Exact or approximate P value?	Approximate
P value summary	****
Do the medians vary signif. (P < 0.05)?	Yes
Number of groups	4
Kruskal-Wallis statistic	35.16
Data summary	
Number of treatments (columns)	4
Number of values (total)	48

Number of families	1					
Number of comparisons per family	3					
Alpha	0.05					
Dunn's multiple comparisons test	Mean rank diff.	Significant?	Summary	Adjusted P Value		
preincubation preultrasound vs. 2h incubation 37 °C	-10.67	No	ns	0.1860	A-B	
2h incubation 37 °C vs. US 2h incubation 37 °C	-6.500	No	ns	0.7662	B-E	
US preincubation preultrasound vs. US 2h incubation 37 °C	-31.67	Yes	****	<0.0001	D-E	
Test details	Mean rank 1	Mean rank 2	Mean rank diff.	n1	n2	Z
preincubation preultrasound vs. 2h incubation 37 °C	21.17	31.83	-10.67	12	12	1.866
2h incubation 37 °C vs. US 2h incubation 37 °C	31.83	38.33	-6.500	12	12	1.137
US preincubation preultrasound vs. US 2h incubation 37 °C	6.667	38.33	-31.67	12	12	5.541

Fig. 4B

Test if UTMD increases number of bacteria indicating dispersion of aggregates

CFU/mL

TSB	TSB+US+MB		pSynF	pSynF+US	pSynF+US+MB		20%p SynF	20% pSynF+US	20% p^{ns}SynF+US+MB
2320000	3840000		34300	48600	43800		46000	70000	66000
2640000	2300000		43200	33600	36600		56000	62000	54000
2040000	2750000		33500	37500	34800		105000	44000	70000
3900000	1750000		39000	28000	37200		80000	50000	77500
2650000	3000000		20500	21500	20000		90000	87000	52000
1320000	3780000		17200	18800	18400		108000	99000	96000
2150000	3100000		17200	28000	16800		69000	92500	107500
1750000	4380000		28500	21000	26400		147000	84000	82500
1980000	3360000		32000	21500	15000		96000	67500	105000
2600000	3060000		27600	18000	17600		105000	67500	62000
3060000	2500000		25000	20000	12800		108000	126000	129000
1100000	2700000		20400	14800	16400		141000	52000	120000
			25500				70000		
			43800				62000		
			37800				123000		
			38400				34000		
			12400				66000		
			14400				74000		
			16400				213000		
			21000				95000		
			24000				111000		
			36600				99000		
			22000				75000		
			28200				54000		

Normality and lognormality

	TSB	TSB+US+MB	pSynF	pSynF+US	pSynF+US+MB	20% pSynF	20% pSynF+US	20% pSynF+US+MB
Test for normal distribution								
D'Agostino & Pearson test								
K2	1.179	0.1088	2.755	5.174	2.470	11.79	1.799	1.593
P value	0.5545	0.9470	0.2522	0.0752	0.2908	0.0028	0.4067	0.4508
Passed normality test (alpha=0.05)?	Yes	Yes	Yes	Yes	Yes	No	Yes	Yes
P value summary	ns	ns	ns	ns	ns	**	ns	ns
Anderson-Darling test								
A2*	0.2050	0.1549	0.3124	0.6325	0.7618	0.5295	0.2627	0.2783
P value	0.8326	0.9382	0.5260	0.0750	0.0340	0.1585	0.6340	0.5824
Passed normality test (alpha=0.05)?	Yes	Yes	Yes	Yes	No	Yes	Yes	Yes
P value summary	ns	ns	ns	ns	*	ns	ns	ns
Shapiro-Wilk test								
W	0.9703	0.9868	0.9591	0.8776	0.8636	0.9150	0.9487	0.9408
P value	0.9140	0.9984	0.4208	0.0816	0.0542	0.0453	0.6181	0.5080
Passed normality test (alpha=0.05)?	Yes	Yes	Yes	Yes	Yes	No	Yes	Yes
P value summary	ns	ns	ns	ns	ns	*	ns	ns
Kolmogorov-Smirnov test								
KS distance	0.1525	0.1355	0.09641	0.2585	0.2522	0.1509	0.1693	0.1366
P value	>0.1000	>0.1000	>0.1000	0.0260	0.0338	>0.1000	>0.1000	>0.1000
Passed normality test (alpha=0.05)?	Yes	Yes	Yes	No	No	Yes	Yes	Yes
P value summary	ns	ns	ns	*	*	ns	ns	ns
Number of values	12	12	24	12	12	24	12	12

Because TSB only had two groups, had to use t test…Mann-Whitney u test for non-parametric data

Table Analyzed	UTMD dilution TSB pSynF
Column B	TSB+US+MB
vs.	vs.
Column A	TSB
Mann Whitney test	
P value	0.0196
Exact or approximate P value?	Exact
P value summary	*
Significantly different (P < 0.05)?	Yes
One- or two-tailed P value?	Two-tailed
Sum of ranks in column A,B	110 , 190
Mann-Whitney U	32
Difference between medians	
Median of column A	2235000, n=12
Median of column B	3030000, n=12
Difference: Actual	795000
Difference: Hodges-Lehmann	730000

Three groups and want inter-group comparisons, so use ANOVA with Kruskal-Wallis test

100% pSynF

Table Analyzed	UTMD dilution TSB pSynF
Kruskal-Wallis test	
P value	0.5228
Exact or approximate P value?	Approximate
P value summary	ns
Do the medians vary signif. (P < 0.05)?	No
Number of groups	3
Kruskal-Wallis statistic	1.297
Data summary	
Number of treatments (columns)	3
Number of values (total)	48

Number of families	1					
Number of comparisons per family	3					
Alpha	0.05					
Dunn's multiple comparisons test	Mean rank diff.	Significant?	Summary	Adjusted P Value		
pSynF vs. pSynF+US	2.208	No	ns	>0.9999	D-E	
pSynF vs. pSynF+US+MB	5.625	No	ns	0.7670	D-F	
pSynF+US vs. pSynF+US+MB	3.417	No	ns	>0.9999	E-F	
Test details	Mean rank 1	Mean rank 2	Mean rank diff.	n1	n2	Z
pSynF vs. pSynF+US	26.46	24.25	2.208	24	12	0.4462
pSynF vs. pSynF+US+MB	26.46	20.83	5.625	24	12	1.137
pSynF+US vs. pSynF+US+MB	24.25	20.83	3.417	12	12	0.5979

20% pSynF

Table Analyzed	UTMD dilution TSB pSynF
Kruskal-Wallis test	
P value	0.3041
Exact or approximate P value?	Approximate
P value summary	ns

Do the medians vary signif. (P < 0.05)?	No
Number of groups	3
Kruskal-Wallis statistic	2.381
Data summary	
Number of treatments (columns)	3
Number of values (total)	48

Number of families	1					
Number of comparisons per family	3					
Alpha	0.05					
Dunn's multiple comparisons test	Mean rank diff.	Significant?	Summary	Adjusted P Value		
20% pSynF vs. 20% pSynF+US	7.625	No	ns	0.3697	H-I	
20% pSynF vs. 20% pSynF+US+MB	2.208	No	ns	>0.9999	H-J	
20% pSynF+US vs. 20% pSynF+US+MB	-5.417	No	ns	>0.9999	I-J	
Test details	Mean rank 1	Mean rank 2	Mean rank diff.	n1	n2	Z
20% pSynF vs. 20% pSynF+US	26.96	19.33	7.625	24	12	1.541
20% pSynF vs. 20% pSynF+US+MB	26.96	24.75	2.208	24	12	0.4464
20% pSynF+US vs. 20% pSynF+US+MB	19.33	24.75	-5.417	12	12	0.9482

Fig. 4C

Comparison of effects of high insonation in the presence of microbubbles.

CFU/ml (trypsin)

TSB

no µbubbles	30 ug/ml AMK	µbubbles	MBL 30 ug/ml AMK	US no µbubbles	US 30 ug/ml AMK	US µbubbles	US MBL 30 ug/ml AMK
7e+008	1400	4.9e+008	1080	4.1e+008	1840	1.82e+007	4280
6.3e+008	1200	8.9e+008	960	2.4e+008	2960	1.97e+007	4650
5.1e+008	1360	1.03e+009	920	3.2e+008	3550	1.12e+007	5080
6e+008		6e+008	1200	3.7e+008	3550	5.22e+007	5640
4.5e+008	470	2.4e+008	260	1.07e+009	120	6.25e+007	3750
4.1e+008	530	5.9e+008	600	1.08e+009	1	6.24e+007	5050
5.2e+008	880	7.2e+008	230	7.2e+008	1	4.4e+007	1760
4.5e+008	540	8.8e+008	240	7.6e+008	1	6.84e+007	1750
6.9e+008	640	6.2e+008	370	1.28e+009	360	4.32e+007	20
5.4e+008		8.8e+008	260	1.12e+009	410	5.34e+007	50
4e+008	840	6.8e+008	510	1.18e+009	540	5.34e+007	1
4.1e+008	800	7.8e+008	460	4.8e+008	270	3.9e+008	1

Normality and lognormality

	no µbubbles	30 ug/ml AMK	µbubbles	MBL 30 ug/ml AMK	US no µbubbles	US 30 ug/ml AMK	US µbubbles	US MBL 30 ug/ml AMK
Test for normal distribution								
D'Agostino & Pearson test								
K2	1.767	1.657	1.548	2.532	5.073	2.961	31.11	6.505
P value	0.4134	0.4368	0.4612	0.2819	0.0791	0.2276	<0.0001	0.0387
Passed normality test (alpha=0.05)?	Yes	Yes	Yes	Yes	Yes	Yes	No	No
P value summary	ns	ns	ns	ns	ns	ns	****	*
Anderson-Darling test								
A2*	0.3941	0.4346	0.2451	0.6366	0.5374	1.323	2.560	0.7506
P value	0.3169	0.2375	0.6973	0.0731	0.1321	0.0011	<0.0001	0.0364
Passed normality test (alpha=0.05)?	Yes	Yes	Yes	Yes	Yes	No	No	No
P value summary	ns	ns	ns	ns	ns	**	****	*
Shapiro-Wilk test								
W	0.9085	0.8935	0.9610	0.8679	0.8942	0.7497	0.5001	0.8434
P value	0.2042	0.1854	0.7976	0.0615	0.1334	0.0027	<0.0001	0.0305
Passed normality test (alpha=0.05)?	Yes	Yes	Yes	Yes	Yes	No	No	No
P value summary	ns	ns	ns	ns	ns	**	****	*
Kolmogorov-Smirnov test								
KS distance	0.1754	0.1838	0.1365	0.1730	0.2146	0.3272	0.4356	0.2068
P value	>0.1000	>0.1000	>0.1000	>0.1000	>0.1000	0.0009	<0.0001	>0.1000
Passed normality test (alpha=0.05)?	Yes	Yes	Yes	Yes	Yes	No	No	Yes
P value summary	ns	ns	ns	ns	ns	***	****	ns
Number of values	12	10	12	12	12	12	12	12

Because of artifact with “1”s in Kruskal-Wallis, did limited number of paired Mann-Whitney tests

Table Analyzed	All TSB for statistics				
	B vs A	E vs D	H vs G	K vs J	H vs B
Column	30 ug/ml AMK	MBL 30 ug/ml AMK	US 30 ug/ml AMK	US MBL 30 ug/ml AMK	US 30 ug/ml AMK
vs.	vs.	vs.	vs.	vs.	vs.
Column	no µbubbles	µbubbles	US no µbubbles	US µbubbles	30 ug/ml AMK

Mann Whitney test					
P value	<0.0001	<0.0001	<0.0001	<0.0001	0.2604
Exact or approximate P value?	Exact	Exact	Exact	Exact	Exact
P value summary	****	****	****	****	ns
Significantly different (P < 0.05)?	Yes	Yes	Yes	Yes	No
One- or two-tailed P value?	Two-tailed	Two-tailed	Two-tailed	Two-tailed	Two-tailed
Sum of ranks in column A,B	198 , 55	222 , 78	222 , 78	222 , 78	132.5 , 120.5
Mann-Whitney U	0	0	0	0	42.5
Difference between medians					
Median of column A	515000000, n=12	700000000, n=12	740000000, n=12	52800000, n=12	820.0, n=10
Median of column B	820.0, n=10	485.0, n=12	385.0, n=12	2755, n=12	385.0, n=12
Difference: Actual	-514999180	-699999515	-739999615	-52797245	-435
Difference: Hodges-Lehmann	-514999065	-699999285	-739998225	-52797180	-380

20% SynF

no µbubbles	30 ug/ml AMK		µbubbles	MBL 30 ug/ml AMK		US no µbubbles	US 30 ug/ml AMK		US µbubbles	US MBL 30 ug/ml AMK
2.3e+007	312000		1.7e+007	96000		2e+007	1		4.84e+007	1
2.85e+007	47000		4e+007	30000		1.2e+007	1		3.8e+007	1
3.4e+007	9700000		3.05e+007	432000		1.2e+007	1		2.84e+007	1
1.24e+007	7000000		2.24e+007	610		1.6e+007	1		4.1e+007	1
2.36e+007	1.28e+007		5.25e+007	1.03e+007		3.04e+007	1		3.12e+007	1
4.08e+007	3100000		3.75e+007	6100000		2.44e+007	1		4.08e+007	1
2.8e+007	1.4e+007		3.66e+007	4500000		1.72e+007	1		2.86e+007	1
3.15e+007	2320		4.04e+007	345000		1.9e+007	1		3.24e+007	1
2.32e+007	2880		3.1e+007	570		3.15e+007	90		4.04e+007	120
2.3e+007	2340		4.44e+007	2580		4.1e+007	120		5.35e+007	70
4.86e+007	4140		4.5e+007	1020		3.72e+007	100		6.7e+007	160
	1.4e+007		4.85e+007	210		3.64e+007	50		5.75e+007	130

Normality and lognormality

	no µbubbles	30 ug/ml AMK	µbubbles	MBL 30 ug/ml AMK	US no µbubbles	US 30 ug/ml AMK	US µbubbles	US MBL 30 ug/ml AMK
Test for normal distribution								
D'Agostino & Pearson test								
K2	1.470	4.103	0.8446	11.41	2.552	3.276	1.522	3.200
P value	0.4796	0.1285	0.6555	0.0033	0.2791	0.1944	0.4672	0.2019
Passed normality test (alpha=0.05)?	Yes	Yes	Yes	No	Yes	Yes	Yes	Yes
P value summary	ns	ns	ns	**	ns	ns	ns	ns
Anderson-Darling test								
A2*	0.3801	1.124	0.2194	2.102	0.3752	1.878	0.3686	1.871
P value	0.3378	0.0037	0.7873	<0.0001	0.3539	<0.0001	0.3676	<0.0001
Passed normality test (alpha=0.05)?	Yes	No	Yes	No	Yes	No	Yes	No
P value summary	ns	**	ns	****	ns	****	ns	****
Shapiro-Wilk test								
W	0.9438	0.7755	0.9646	0.6268	0.9211	0.6770	0.9227	0.6791
P value	0.5665	0.0050	0.8474	0.0002	0.2953	0.0005	0.3089	0.0005
Passed normality test (alpha=0.05)?	Yes	No	Yes	No	Yes	No	Yes	No
P value summary	ns	**	ns	***	ns	***	ns	***
Kolmogorov-Smirnov test								
KS distance	0.1872	0.2858	0.1457	0.4099	0.1794	0.4052	0.2081	0.4063
P value	>0.1000	0.0076	>0.1000	<0.0001	>0.1000	<0.0001	>0.1000	<0.0001

Passed normality test (alpha=0.05)?	Yes	No	Yes	No	Yes	No	Yes	No
P value summary	ns	**	ns	****	ns	****	ns	****
Number of values	11	12	12	12	12	12	12	12

Because of artifact with "1"s in Kruskal-Wallis, did limited number of paired Mann-Whitney tests

Table Analyzed	All 20% hSynF for statistics				
	B vs A	E vs D	H vs G	K vs J	H vs B
Column	30 ug/ml AMK	MBL 30 ug/ml AMK	US 30 ug/ml AMK	US MBL 30 ug/ml AMK	US 30 ug/ml AMK
vs.	vs.	vs.	vs.	vs.	vs.
Column	no µbubbles	µbubbles	US no µbubbles	US µbubbles	30 ug/ml AMK
Mann Whitney test					
P value	<0.0001	<0.0001	<0.0001	<0.0001	<0.0001
Exact or approximate P value?	Exact	Exact	Exact	Exact	Exact
P value summary	****	****	****	****	****
Significantly different (P < 0.05)?	Yes	Yes	Yes	Yes	Yes
One- or two-tailed P value?	Two-tailed	Two-tailed	Two-tailed	Two-tailed	Two-tailed
Sum of ranks in column A,B	195 , 81	222 , 78	222 , 78	222 , 78	222 , 78
Mann-Whitney U	3	0	0	0	0
Difference between medians					
Median of column A	28000000, n=11	38750000, n=12	22200000, n=12	40600000, n=12	1706000, n=12
Median of column B	1706000, n=12	63000, n=12	1.000, n=12	1.000, n=12	1.000, n=12
Difference: Actual	-26294000	-38687000	-22199999	-40599999	-1705999
Difference: Hodges-Lehmann	-23075340	-37111500	-22199940	-40599920	-1705940

Fig. 5E

Histology data

Hyperplasia AMK	Hyperplasia AMK+UTMD	Inflammatory Infiltrate AMK	Inflammatory Infiltrate AMK+UTMD	Synovial Stromal Activity AMK	Synovial Stromal Activity AMK+UTMD	Total Score AMK	Total Score AMK+UTMD
1	2	1	3	1	2	3	7
	2		3		3		8
3	3	3	3	3	3	9	9
1		1		1		3	
3	2	3	3	3	3	9	8
	2		3		3		8
	2		1		1		4
	1		1		1		3
	3		2		3		8

	Hyperplasia AMK	Hyperplasia AMK+UTMD	Inflammatory Infiltrate AMK	Inflammatory Infiltrate AMK+UTMD	Synovial Stromal Activity AMK	Synovial Stromal Activity AMK+UTMD	Total Score AMK	Total Score AMK+UTMD
Test for normal distribution								
Kolmogorov-Smirnov test								
KS distance	N too small	0.3273	N too small	0.3775	N too small	0.3775	N too small	0.3232
P value		0.0117		0.0013		0.0013		0.0138
Passed normality test (alpha=0.05)?		No		No		No		No
P value summary		*		**		**		*
Number of values	4	8	4	8	4	8	4	8

Table Analyzed	Histology Scores
Kruskal-Wallis test	
P value	0.0014
Exact or approximate P value?	Approximate
P value summary	**
Do the medians vary signif. (P < 0.05)?	Yes
Number of groups	8
Kruskal-Wallis statistic	23.48
Data summary	
Number of treatments (columns)	8
Number of values (total)	48

Number of families	1					
Number of comparisons per family	4					
Alpha	0.05					
Dunn's multiple comparisons test	Mean rank diff.	Significant?	Summary	Adjusted P Value		
Hyperplasia AMK vs. Hyperplasia AMK+UTMD	0.1250	No	ns	>0.9999	A-B	
Inflammatory Infiltrate AMK vs. Column D	-4.000	No	ns	>0.9999	C-D	
Synovial Stromal Activity AMK vs. Column F	-4.000	No	ns	>0.9999	E-F	
Total Score AMK vs. Total Score AMK+UTMD	-3.375	No	ns	>0.9999	G-H	
Test details	Mean rank 1	Mean rank 2	Mean rank diff.	n1	n2	Z
Hyperplasia AMK vs. Hyperplasia AMK+UTMD	17.50	17.38	0.1250	4	8	0.01536
Inflammatory Infiltrate AMK vs. Column D	17.50	21.50	-4.000	4	8	0.4916
Synovial Stromal Activity AMK vs. Column F	17.50	21.50	-4.000	4	8	0.4916
Total Score AMK vs. Total Score AMK+UTMD	38.00	41.38	-3.375	4	8	0.4148

CFU from PIG (colony counts)

AMK

Pre-TX	Post-TX
11000	8200
2200	5050
800	1400
1400	270
12500	9750

	Pre-TX	Post-TX
Test for normal distribution		
Kolmogorov-Smirnov test		
KS distance	0.3241	0.2041
P value	0.0931	>0.1000
Passed normality test (alpha=0.05)?	Yes	Yes
P value summary	ns	ns
Number of values	5	5

Table Analyzed	Consolidated pig exp. AMK
Column B	Post-TX
vs.	vs.
Column A	Pre-TX
Mann Whitney test	
P value	0.7381
Exact or approximate P value?	Exact
P value summary	ns
Significantly different (P < 0.05)?	No
One- or two-tailed P value?	Two-tailed
Sum of ranks in column A,B	29.50 , 25.50
Mann-Whitney U	10.50
Difference between medians	
Median of column A	2200, n=5
Median of column B	5050, n=5
Difference: Actual	2850
Difference: Hodges-Lehmann	-800.0

AMK+US

Pre-TX	Post-TX
800	7390
1500	6708
18500	8250

	Pre-TX	Post-TX
Test for normal distribution		
Kolmogorov-Smirnov test		
KS distance	N too small	N too small
P value		
Passed normality test (alpha=0.05)?		
P value summary		
Number of values	3	3

Table Analyzed	Consolidated pig exp. AMK+U/S
Column B	Post-TX
vs.	vs.
Column A	Pre-TX
Mann Whitney test	
P value	0.7000
Exact or approximate P value?	Exact
P value summary	ns
Significantly different (P < 0.05)?	No
One- or two-tailed P value?	Two-tailed
Sum of ranks in column A,B	9 , 12
Mann-Whitney U	3
Difference between medians	
Median of column A	1500, n=3
Median of column B	7390, n=3
Difference: Actual	5890
Difference: Hodges-Lehmann	5890

AMK + microbubbles

Pre-TX	Post-TX
2800	1
2500	1
1	1
1590	1.1
1400	1
2230	1.1
1250	1.1

	Pre-TX	Post-TX
Test for normal distribution		
Kolmogorov-Smirnov test		
KS distance	0.1805	0.3601
P value	>0.1000	0.0065
Passed normality test (alpha=0.05)?	Yes	No
P value summary	ns	**
Number of values	7	7

Table Analyzed	Consolidated pig exp. AMK+MCBL
Column B	Post-TX
vs.	vs.
Column A	Pre-TX
Mann Whitney test	
P value	0.0099
Exact or approximate P value?	Exact
P value summary	**
Significantly different (P < 0.05)?	Yes
One- or two-tailed P value?	Two-tailed
Sum of ranks in column A,B	72 , 33
Mann-Whitney U	5
Difference between medians	
Median of column A	1590, n=7
Median of column B	1.000, n=7
Difference: Actual	-1589

Difference: Hodges-Lehmann	-1589
